# Screening of Phosphodiesterase-5 Inhibitors and Their Analogs in Dietary Supplements by Liquid Chromatography–Hybrid Ion Trap–Time of Flight Mass Spectrometry

**DOI:** 10.3390/molecules25122734

**Published:** 2020-06-12

**Authors:** Unyong Kim, Hyun-Deok Cho, Myung Hee Kang, Joon Hyuk Suh, Han Young Eom, Junghyun Kim, Sumin Seo, Gunwoo Kim, Hye Ryoung Koo, Nary Ha, Un Tak Song, Sang Beom Han

**Affiliations:** 1 Department of Pharmaceutical Analysis, College of Pharmacy, Chung-Ang University, 84 Heukseok-ro, Dongjak-gu, Seoul 06974, Korea; unyong.kim@biocomplete.co.kr (U.K.); green007n@daum.net (H.-D.C.); ssm1302@naver.com (S.S.); poyoo65@naver.com (G.K.); hrcecilia@naver.com (H.R.K); nrha@yuhan.co.kr (N.H.); utsong@shimadzu.co.kr (U.T.S.); 2 Biocomplete Co., Ltd., 272 Digital-ro, Guro-gu, Seoul 08389, Korea; 3 Bioanalysis and Pharmacokinetics Study Group, Korea Institute of Toxicology, 141 Gajeong-ro, Yuseong-gu, Daejeon 34114, Korea; hanyoung.eom@kitox.re.kr; 4 Agro-Livestock and Fishery Products Division, Busan Regional Korea Food and Drug Administration, 222 Geoje-daero, Yunje-gu, Busan 47537, Korea; kangmh68@korea.kr; 5 Department of Food Science and Human Nutrition, Citrus Research and Education Center, University of Florida, 700 Experiment Station Rd, Lake Alfred, FL 33850, USA; joonhyuksuh@ufl.edu; 6 Forensic Toxicology Division, National Forensic Service, 10 Ipchoon-ro, Wonju, Gangwon-do 26460, Korea; jhkim1807@korea.kr

**Keywords:** phosphodiesterase-5 inhibitors, analogs, dietary supplements, high-performance liquid chromatography, hybrid ion trap–time of flight mass spectrometry

## Abstract

An accurate and reliable method based on ion trap–time of flight mass spectrometry (IT–TOF MS) was developed for screening phosphodiesterase-5 inhibitors, including sildenafil, vardenafil, and tadalafil, and their analogs in dietary supplements. Various parameters affecting liquid chromatographic separation and IT–TOF detection were investigated, and the optimal conditions were determined. The separation was achieved on a reversed-phase column under gradient elution using acetonitrile and water containing 0.2% acetic acid at a flow rate of 0.2 mL/min. The chromatographic eluents were directly ionized in the IT–TOF system equipped with an electrospray ion source operating in the positive ion mode. The proposed screening method was validated by assessing its linearity, precision, and accuracy. Sequential tandem MS was conducted to obtain structural information of the references, and the fragmentation mechanism of each reference was proposed for providing spectral insight for newly synthesized analogs. Structural information, including accurate masses of both parent and fragment ions, was incorporated into the MS^n^ spectral library. The developed method was successfully applied for screening adulterated dietary supplement samples.

## 1. Introduction

Penile erection is started from the sexual stimulation which causes the release of nitric oxide (NO) from nerves and cells in the penis. The released NO diffuses into the cells and activates guanylyl cyclase synthesizing cyclic GMP (cGMP). cGMPs, an intra-cellular second messenger, cause the smooth muscle of corpus cavernosum to relax, which in turn promotes inflow of blood, and ultimately, compression of the spongy corpus cavernosum tissue. This compression results in penile erection [1]. Phosphodiesterase type 5 (PDE-5) is a primary hydrolytic enzyme that is localized mainly in the human corpus cavernosum tissue. It plays a major biological role in the hydrolysis of cyclic guanosine monophosphate (cGMP) to 5′-GMP. PDE-5 inhibitors such as sildenafil (Viagra, Pfizer, New York, NY, USA), vardenafil (Levitra, Bayer, Leverkusen, Germany), tadalafil (Cialis, Elli Lilly, Indianapolis, IN, USA), mirodenafil (Mvix, SK Life Science, Seongnam, Republic of Korea), and udenafil (Zydena, Dong-A Pharmaceutical, Seoul, Republic of Korea) compete with cGMP for the catalytic sites composed of 16 helices and 16 loops at the C-terminal end (amino acid residues:535–860) [2] of the PDE-5 enzyme and retard the enzymatic hydrolysis of cGMP in the corpus cavernosum, which eventually leads to penile erection [1]. Thus, PDE-5 inhibitors are used for the treatment of erectile dysfunction (ED).

Patients over the age of 40 suffering from chronic diseases such as hypertension, ischemic heart disease, depression, diabetes, and atherosclerosis commonly complain of ED as a complication [1]. However, the drugs for the treatment of these chronic diseases, such as nitroglycerin, doxazosin, and terazosin, interact negatively with PDE-5 inhibitors [3]. This negative aspect of synthetic PDE-5 inhibitors has triggered the development of herbal alternatives. Thus, herbal therapies have successfully captured the market because most people believe that they are safe from adverse effects, and they can be purchased without any prescription [3,4,5]. However, taking advantage of the recognition that natural products are safe, illegally adding synthetic PDE-5 inhibitors and their analogs to herbal dietary supplements or making counterfeit drugs began to be distributed [6,7,8,9,10,11,12]. Since the detection of the first sildenafil analog, homosildenafil, in dietary supplements, the number of analogs has steadily increased owing to various structural modifications of the original drugs [5,6].

As the structure of these analogs is similar to that of the original drugs, their pharmacological effects are similar to those of the final developed drugs except for their potency, side effects, and toxicity. Therefore, the adulterated analogs in dietary supplements may pose significant pharmacological and toxicological risks such as headaches, dyspepsia, myalgia, flushing, dizziness, and abnormal vision [5,13]. For example, hongdenafil analogs cause visual disturbances even in low doses because hongdenafil lacks PDE-5/PDE-6 selectivity [5,14,15]. In addition, aminotadalafil has a reactive hydrazone group that permanently inhibits the enzymes [5,14]. Therefore, undeclared PDE-5 inhibitors and their analogs in dietary supplements threaten public health.

The analytical methods for determining undeclared PDE-5 inhibitors and their analogs in dietary supplements need to be developed for public health safety and regulation. In this regard, various spectrometric and chromatographic methods have been adopted to analyze the illegally added drugs in complex matrices [3,4,5,9,16,17,18,19,20,21,22,23,24,25,26,27,28,29]. High-performance liquid chromatography with diode array detection (HPLC–DAD) and liquid chromatography with mass spectrometry (LC–MS) have been previously used to detect PDE-5 inhibitors and their analogs [3,5,7,8,9,10,16,17,20,21,22,23,24,25,26,27,28,29,30,31,32,33,34]. When the standards of PDE-5 inhibitors and their analogs are available, HPLC with a UV spectrometer is a simple method for quantitative analysis [11]. In HPLC–DAD, the analytes can be identified by comparing the UV spectra of the reference compounds and analytes of interest [3,10,17,33]. In this context, MS has attracted considerable interest for both qualitative and quantitative analysis. Particularly in quantitative analysis, multiple reaction monitoring (MRM) in triple quadrupole mass spectrometry (QqQ MS) shows unique selectivity with high sensitivity. Thus, QqQ MS is usually used to detect analytes in complex matrices using MRM with a unique ion transition of the analytes [16,20,22,32]. Although DAD provides the UV spectra of certain compounds identified in a given chromatogram, there are no authentic UV spectra of newly synthesized PDE-5 inhibitors. Further, QqQ MS can only be used for the quantitative analysis of known compounds and not for screening purposes. Therefore, other spectroscopic methods such as high-resolution mass spectrometry (HRMS) based on Fourier transform ion cyclotron resonance (FT-ICR) [31], time of flight (TOF) [24,25,28,29,33], orbitrap [8,12,26,27], and nuclear magnetic resonance spectrometry [4,6,7,9,30,34,35,36,37,38] must be applied to clarify the structure of the analytes. In particular, hybrid ion trap (IT)–HRMS, including IT–TOF and Orbitraps (LTQ–Orbitrap and Orbitrap Fusions), is one of the most effective tools for screening and identification of unknown compounds owing to its high-resolution and multistage tandem MS (MS^n^) ability. The MS^n^ ability of the IT–HRMS provides more structural information than does QqQ MS by sequential trapping and fragmentation of the precursor ion, and accurate masses of both parent and fragment ions can be obtained by HRMS for further conversion into elemental composition.

In this study, we developed a screening method for adulterated PDE-5 inhibitors and their analogs in dietary supplements by using HPLC coupled to IT–TOF MS. The HPLC separation conditions such as mobile phase and gradient, and MS parameters were optimized for the best chromatographic resolution and for obtaining tandem MS spectra with rich information about the parent and fragment ions. In addition, a spectral library for 38 PDE-5 inhibitors and their analogs was built using the high-resolution tandem MS spectra obtained in this study. The developed method was applied to dietary supplements that were adulterated with PDE-5 inhibitors and their analogs. We could successfully identify the illegally fortified PDE-5 inhibitors using the spectral library.

## 2. Results and Discussion

### 2.1. HPLC–MS Method Development

As the selected precursor ions for MS^n^ analysis are isolated and undergo collision-induced dissociation (CID) in IT MS, wherein the analysis is performed at the unit mass resolution, chromatographic separation is prerequisite for the simultaneous qualification of isobaric compounds. In this study, different stationary phases were tested to optimize the separation of isobaric compounds from mixtures of 38 standards of PDE-5 inhibitors and their analogs. The separation of isobaric compounds among three different stationary phases was compared. The Capcell PAK C18 column showed the best chromatographic separation performance and was hence selected for LC–MS analysis.

Various mobile phase additives, including formic acid, acetic acid, ammonium formate, and ammonium acetate, were investigated for the separation of isobaric compounds. Although formic acid, ammonium formate, and ammonium acetate showed better separation than acetic acid, they negatively affected the MS sensitivity. Sensitivity, which is mainly affected by the ionization conditions, is imperative for the identification of unknown compounds in MS^n^ analysis. Thus, acetic acid was chosen as the mobile phase additive to improve the MS sensitivity. Between two different concentrations (0.1% and 0.2%) of acetic acid, 0.2% acetic acid gave better separation efficiency. Considering the ionization efficiency and chromatographic separation of isobaric compounds, acetic acid at a concentration of 0.2% (*v*/*v*) was selected as the mobile phase additive.

**Figure 1:**
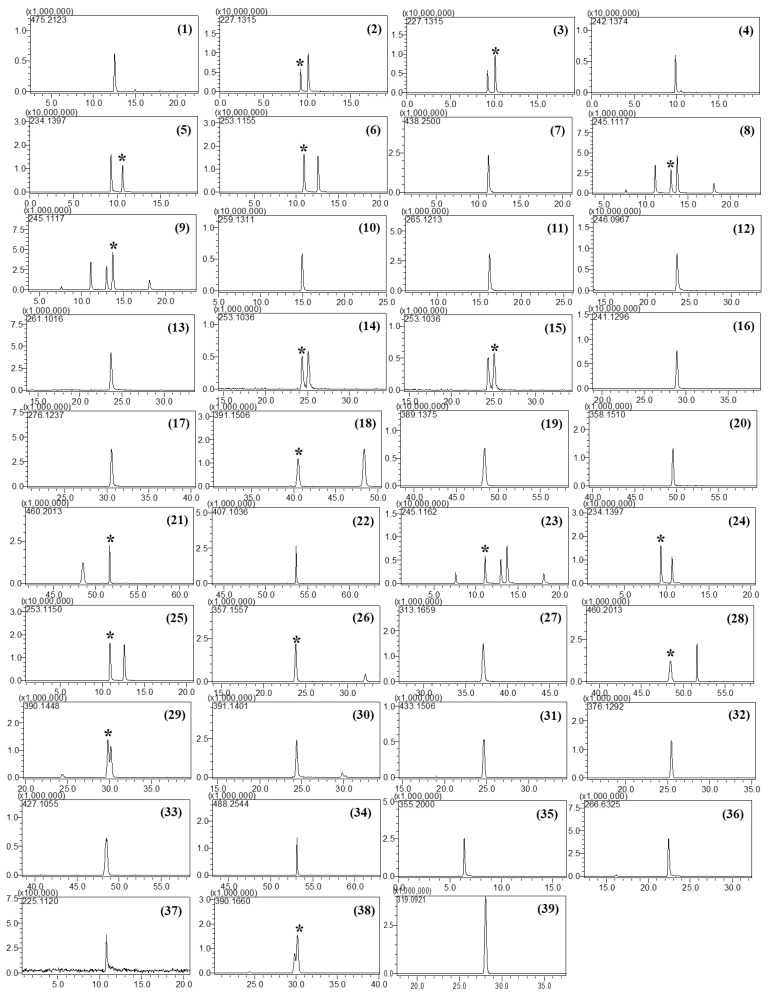
Extracted ion chromatograms of PDE-5 inhibitors and their analogs: Sildenafil (**1**), carbodenafil (**2**), demethylhongdenafil (**3**), hydroxyhongdenafil (**4**), hongdenafil (**5**), hydroxyhomosildenafil (**6**), piperidinohongdenafil (**7**), homosildenafil (**8**), dimethylsildenafil (**9**), udenafil (**10**), cyclopentinafil (**11**), thiosildenafil (**12**), hydroxythiohomosildenafil (**13**), thiohomosildenafil (**14**), dimethylthiosildenafil (**15**), oxohongdenafil (**16**), benzylsildenafil (**17**), hydroxychlorodenafil (**18**), chlorodenafil (**19**), nitrodenafil (**20**), nor-neosildenafil (**21**), dichlorodenafil (**22**), vardenafil (**23**), acetylvardenafil (**24**), hydroxyvardenafil (**25**), nor-neovardenafil (**26**), desulfovardenafil (**27**), pseudovardenafil (**28**), tadalafil (**29**), aminotadalafil (**30**), acetaminotadalafil (**31**), demethyltadalafil (**32**), chloropretadalafil (**33**), *N*-octyltadalafil (**34**), yohimbine (**35**), mirodenafil (**36**), thioquinapiperifil (**37**), xanthoanthrafil (**38**), and phenolphthalein (**39**). * indicated the analytes among multiple peaks in the EIC.

Furthermore, the column temperature was a critical factor that affected the separation of isobaric compounds in this study. Five different temperatures (30, 35, 40, 45, and 50 °C) were tested, and the best separation was achieved at 50 °C. No attempt was made to increase the column temperature above 50 °C as doing so could damage the column. Although some compounds such as tadalafil and xanthoanthrafil did not show baseline separation under the given chromatographic conditions, they could be distinguished by their accurate masses. Accordingly, a temperature of 50 °C was selected for LC–MS analysis. The retention times and mass errors of all analytes are listed in Table 1, and the extracted ion chromatograms are depicted in Figure 1.

### 2.2. Method Validation

As the main purpose of this study was qualitative analysis, validation was limited to verification of the method. Nevertheless, quantitative analysis was required in some cases. To improve the quantitative precision and accuracy of our analytical method, phenolphthalein was used as an internal standard (IS).

At a higher concentration, saturation of the IT occurred, and loss of linearity and mass spectral distortion were observed owing to the space charge effect in the IT-type MS [39]. Thus, linearity was estimated over a narrow range. Each calibration curve was constructed with six different concentrations of the 38 PDE-5 inhibitors and their analogs through linear regression analysis. All compounds showed appropriate linearity (*R*^2^ > 0.99) over the estimated concentration ranges (Table 2).

The precision of the method was evaluated in terms of intra- and inter-day precision, estimated by testing a mixed standard solution in five replicates in a day and by repeating the test on five consecutive days. The QC samples were consisted with three different concentrations: Low concentration (L): the lowest concentration in the calibration curve; Medium concentration (M): about 1.67 times higher concentration than low concentration; High concentration (H): the highest concentration in the calibration curve. The intra- and inter-day precision of the 38 PDE-5 inhibitors and their analogs ranged from 0.6% to 9.2% and from 1.2% to 10.5%, respectively. The corresponding intra- and inter-day accuracy ranged from 86.7% to 112.0% and from 89.7% to 105.7%. Based on the results for the validation parameters, this method was demonstrated to be reproducible and reliable for the tested concentration range (Table 3).

The reproducibility of the accurate mass measurement of the analytes was evaluated by testing a mixed standard solution in six replicates. The accurate masses, the mass errors to theoretical mass, and standard deviation (SD) values are listed in Table 4. The reproducibility of accurate mass measurement ranged from 0.42 to 5.47 as SD, and the mass errors to theoretical mass were less than 10 ppm. As the average mass of carbon varies between 12.0107 and 12.0111 due to variations in the natural abundance of ^13^C, the mass accuracy was limited to 10 ppm [40]. Because the fluctuation of the flight tube temperature could affect the flight distance of the analytes and eventually increase or decrease the mass errors, the temperature of the flight tube in the TOF mass analyzer was kept at 40 °C to maintain the mass accuracy [41]. In addition, the TOF analyzer was calibrated before the analysis using sodium trifluoroacetate solution in accordance with the in-house standard operating procedure.

### 2.3. MS^n^ Analysis

**Figure 2:**
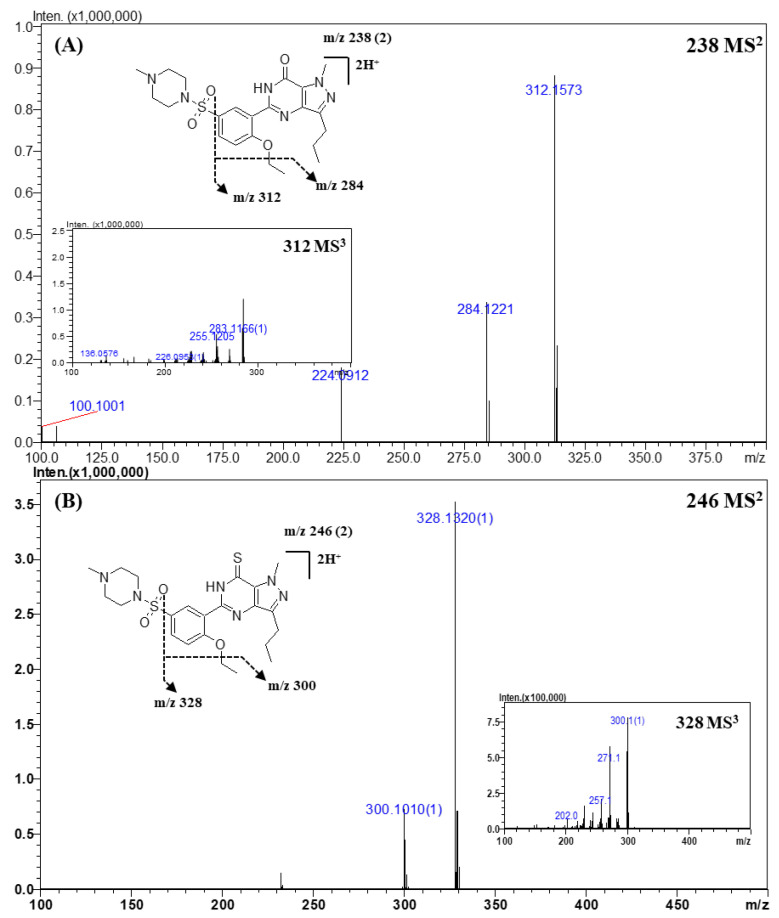
Representative MS^n^ (*n* = 2, 3) spectra and proposed fragmentation mechanisms of sildenafil (**A**) and thiosildenafil (**B**). The bracketed numbers next to the *m*/*z* values indicate the charge state of the ions.

**Figure 3:**
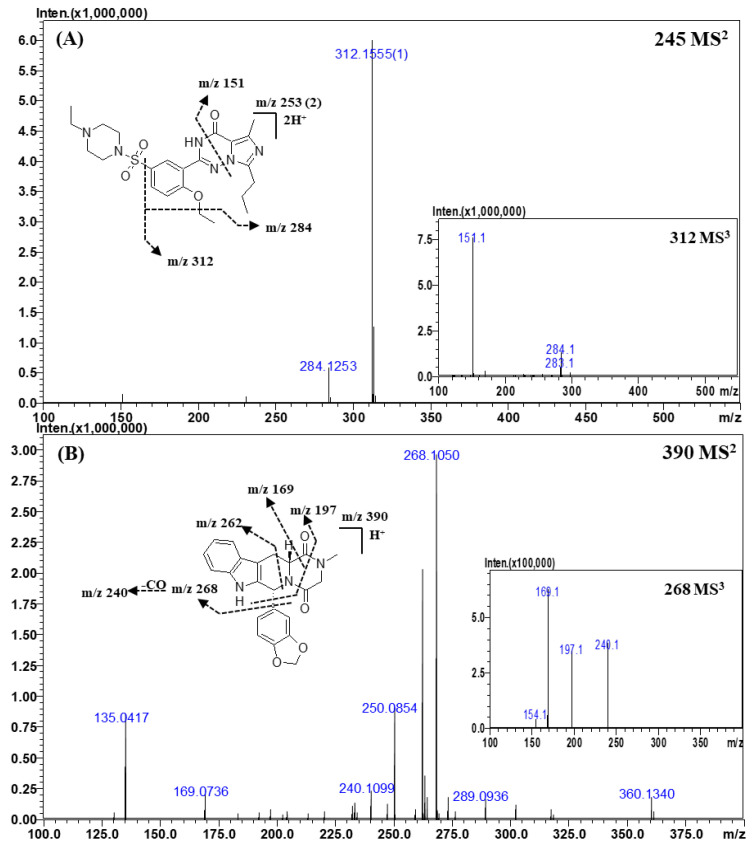
Representative MS^n^ (*n* = 2, 3) spectra and proposed fragmentation mechanisms of vardenafil (**A**) and tadalafil (**B**). The bracketed numbers next to the *m*/*z* values indicate the charge state of the ions.

**Figure 4:**
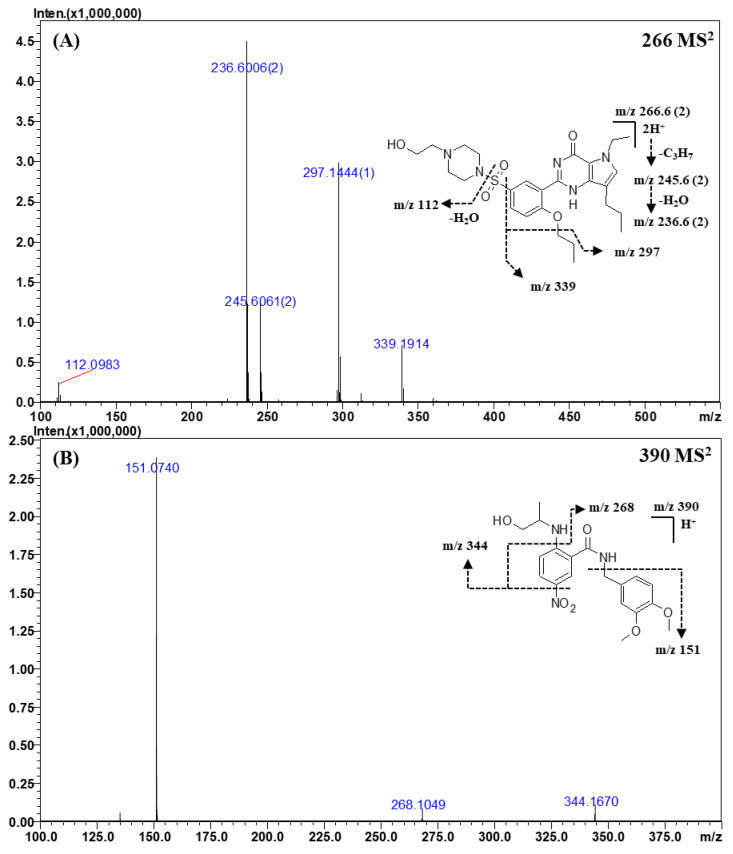
Representative MS^n^ (*n* = 2, 3) spectra and proposed fragmentation mechanisms of mirodenafil (**A**) and xanthoanthrafil (**B**). The bracketed numbers next to the *m*/*z* values indicate the charge state of the ions.

**Figure 5:**
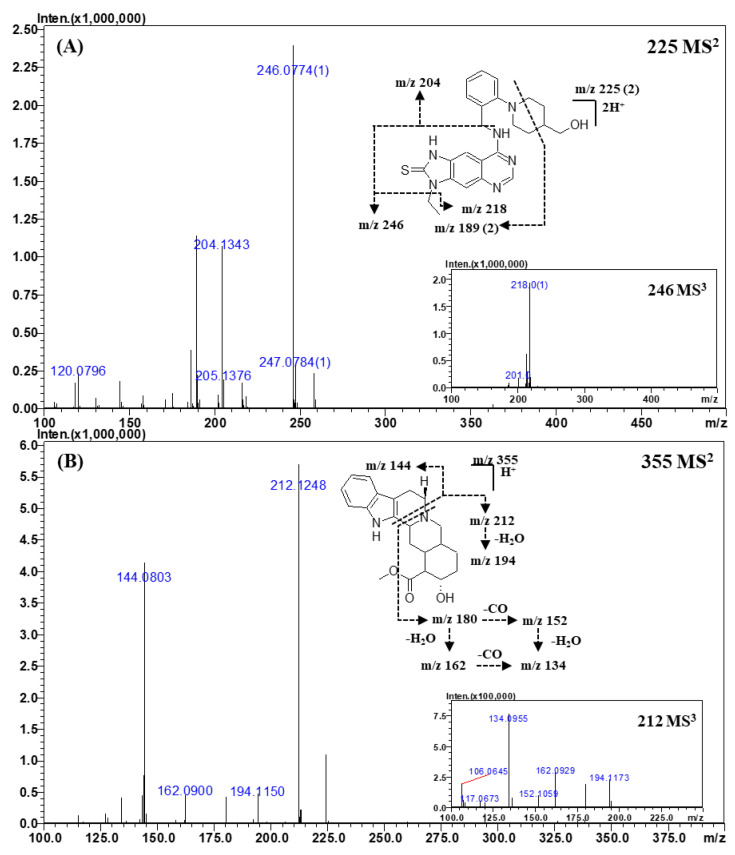
Representative MS^n^ (*n* = 2, 3) spectra and proposed fragmentation mechanisms of thioquinapiperifil (**A**) and yohimbine (**B**). The bracketed numbers next to the *m*/*z* values indicate the charge state of the ions.

For identification, a tandem MS analysis should be performed for both references and the analytes of interests as it allows for a clear comparison of their spectra. To construct the spectral library, the reference spectra should contain as many fragment ions as possible because they can be used as queries. Therefore, optimization of the collision energy is important to build the spectral library. At low collision energy (<150% of the relative collision energy), the tandem MS spectra contained only a limited number of fragment ions, such as the dehydrated (“parent ion—18 Da”) form of the parent ion. At the relative collision energy of 150%, there were more fragment ions as compared to those formed at a lower collision energy without loss of information of the parent ions. Accordingly, the applied relative collision energy was set to 150%. In addition, as some compounds did not produce identifiable fragment ions in MS^2^ analysis, MS^3^ analysis was performed to obtain more fragment ions for more efficient identification and for effectively building the spectral library. The mass errors, elemental compositions, and accurate masses of the precursors and fragment ions are listed in Table 4 and Table S1. The MS^n^ spectra and proposed fragmentation mechanisms are depicted in Figure 2, Figure 3, Figure 4 and Figure 5 and Figures S1–S15.

#### 2.3.1. Sildenafil and Its Derivatives

Representative MS^2^ and MS^3^ spectra, and the proposed fragmentation mechanism of sildenafil and its derivatives are shown in Figure 2. A total of 22 compounds of sildenafil and their derivatives were analyzed. In the CID process, sildenafil and its derivatives showed a common fragmentation mechanism owing to their structural similarity. The exact masses and elemental compositions of the major fragment ions observed in the MS^2^ or MS^3^ spectra were *m*/*z* 312.1573 and *m*/*z* 284.1221, and their elemental compositions were C_17_H_19_N_4_O_2_ and C_15_H_15_N_4_O_2_, respectively (Table 4 and Figure 2A). The product ion of *m*/*z* 312.1573 was formed by a neutral loss of the sulfonyl group, and the ion of *m*/*z* 284.1221 was produced by a neutral loss of an ethyl moiety (-C_2_H_2_, -28 Da) from the ion of *m*/*z* 312.1573. In addition, product ions of *m*/*z* 311.1456 and *m*/*z* 283.1162 had 1 Da lower mass than that of the major fragment ions. The presence of the two groups of fragment ions indicated that more than one pathway could account for the formation of these fragments. The fragment ion observed at *m*/*z* 312.1573 was produced by the homolytic cleavage of the C-S bond, while the ion of *m*/*z* 311.1456 was formed by inductive cleavage [42]. This fragmentation mechanism was the same as that reported in the literature [3,11,31,42].

In the case of thiosildenafil derivatives (Figure 2B), the proposed fragmentation mechanism was almost the same as that described above; fragment ions of *m*/*z* 328.1344 (C_17_H_19_N_4_OS) and *m*/*z* 300.1022 (C_15_H_15_N_4_OS) were formed by C-S bond cleavage and subsequent detachment of the ethyl moiety. The mass of the fragment ions was 16 Da lower than that of the fragments from sildenafil; this difference was attributable to the presence of the thioketone moiety, whose mass was 16 Da higher than that of the ketone moiety in sildenafil.

Isobaric analytes could be distinguished by their retention times and MS^2^ or MS^3^ spectra. For instance, the theoretical mass and elemental compositions of homosildenafil and dimethylsildenafil were 489.2229 Da and C_23_H_32_N_6_O_4_S, respectively; however, their retention times were different (13.0 and 13.7 min, respectively, Figure 1 and Table 2). In the MS^2^ spectra of homosildenafil and dimethylsildenafil (Figure S1), the ion of *m*/*z* 312 was observed as a base peak, and the ion of *m*/*z* 284 was the second most intense peak. The ion ratio of *m*/*z* 284.1230 to *m*/*z* 312.1542 in the MS^2^ spectrum of homosildenafil was about three times higher than that in the spectrum of dimethylsildenafil. Thiohomosildenafil and dimethylthiosildenafil were separately observed at retention times of 24.1 and 25.1 min (Table 2), and their elemental composition was C_23_H_32_N_6_O_3_S_2_. Their MS^2^ and MS^3^ spectra were almost the same, except for their different *m*/*z* 300.1012 to *m*/*z* 328.1332 ion ratios (Figure S2). In the MS^2^ spectrum of thiohomosildenafil, the ion ratio of *m*/*z* 300.1019 to *m*/*z* 328.1364 was approximately three times higher than that in the MS^2^ spectrum of dimethylthiosildenafil. These results indicate that both the separation capability and the ion ratio of certain fragment ions in tandem MS spectra were important to gain sufficient information for discriminating the isobaric compounds.

#### 2.3.2. Vardenafil and Its Derivatives

Representative MS^2^ and MS^3^ spectra and the proposed fragmentation mechanism of vardenafil are shown in Figure 3A. The structures of vardenafil and its derivatives were almost identical to those of sildenafil and its derivatives. In the CID spectra of vardenafil and its derivatives, the fragment ions of *m*/*z* 312.1555 (C_17_H_19_N_4_O_2_) and *m*/*z* 284.1253 (C_15_H_15_N_4_O_2_) were formed by the fragmentation mechanisms identical to those for sildenafil and its derivatives owing to their structural similarity. However, the ions produced by the homolytic cleavage of the C-S bond were not found in the MS^n^ spectra of vardenafil, and the ion of *m*/*z* 151.0877 (C_8_H_10_N_2_O), which was formed by the cross ring cleavage of triazin-4-one, was observed only in the CID process of vardenafil and its derivatives.

Because of the structural similarity between the vardenafil and sildenafil derivatives, the fragment ions generated in the MS^2^ or MS^3^ spectra were almost same. For example, when comparing the MS^2^ spectra of the vardenafil and sildenafil derivatives with the same molecular weights, such as homosildenafil and dimethylsildenafil, the major fragment ions were observed at *m*/*z* 312.1555 and *m*/*z* 284.1253. The ratio of these ions in the MS^2^ spectrum of vardenafil was similar to that in the spectrum of homosildenafil. Therefore, it was difficult to distinguish between vardenafil and homosildenafil by their MS^2^ spectra on the basis of the major fragment ions and the ion ratio of a certain fragment ion. As previously described in this section, the ion of *m*/*z* 151.0877 (C_8_H_10_N_2_O), a diagnostic ion of vardenafil, was only found in the MS^2^ or MS^3^ spectra of vardenafil analogs. Thus, we could distinguish vardenafil analogs from those of sildenafil by simply observing the diagnostic ion in the MS^2^ or MS^3^ spectra of the analytes.

#### 2.3.3. Tadalafil and Its Derivatives

Representative MS^2^ and MS^3^ spectra and the proposed fragmentation mechanism of tadalafil are depicted in Figure 3B. In the CID spectra of tadalafil, the ion observed at *m*/*z* 268.1050 (C_15_H_13_N_3_O_2_) was formed by a neutral loss of 1,3-benzodioxole, and the ion of *m*/*z* 240.1099 (C_14_H_13_N_3_O) was produced by the loss of the carbonyl (CO) group from the ion with *m*/*z* 268.1050. The ion of *m*/*z* 197.0707 (C_12_H_8_N_2_O) was formed by a neutral loss of 1,3-benzodioxole and the cross ring cleavage of piperazine-2,5-dione, and the ion of *m*/*z* 169.0736 (C_11_H_8_N_2_) was produced by the loss of a CO group from the ion of *m*/*z* 197.0707. The ion observed at *m*/*z* 262.0866 was formed by a neutral loss of piperazine-2,5-dione. The fragment ions of *m*/*z* 262.0866, *m*/*z* 197.0707, and *m*/*z* 169.0736 and a neutral loss of 1,3-benzodioxole are commonly observed in the CID spectra of tadalafil derivatives.

#### 2.3.4. Other Classes

The CID spectra of mirodenafil, xanthoanthrafil, thioquinapiperifil, and yohimbine are shown in Figure 4; Figure 5. As the structure of mirodenafil was almost the same as that of sildenafil, its fragment ions were produced by the same fragmentation mechanism as that of sildenafil (Figure 4A). The ion observed at *m*/*z* 339.1914 (C_20_H_24_N_3_O_2_) was formed by the cleavage of the C-S bond, and the ion of *m*/*z* 297.1444 (C_17_H_18_N_3_O_2_) was produced by a neutral loss of the propyl group of phenyl propyl ether. The doubly charged ion of *m*/*z* 245.6061 (C_23_H_31_N_5_O_5_S) was formed by a neutral loss of the propyl group of phenyl propyl ether, and the ion of *m*/*z* 236.6006 (C_23_H_29_N_5_O_4_S) was produced by intra-molecular dehydration (-H_2_O) from the ion of *m*/*z* 245.6061.

The structures of xanthoanthrafil and thioquinapiperifil were different from those of other PDE-5 inhibitors and their analogs. As they have unique structures, the fragment ions produced in the CID spectra were different from those of other classes of analytes.

In the MS^2^ spectra of xanthoanthrafil (Figure 4B), the fragment ion of *m*/*z* 151.0740 (C_9_H_10_O_2_) was predominant. The ion observed at *m*/*z* 151.0740 was formed by a neutral loss of the 3-nitrobenzamide group. The ions of *m*/*z* 344.1670 (C_19_H_23_N_2_O_4_) and *m*/*z* 268.1049 (C_16_H_14_NO_3_) were produced by a sequential neutral loss of the NO_2_ group.

The fragment ions of thioquinapiperifil in the MS^2^ spectra (Figure 5A) were observed at *m*/*z* 246.0774 (C_11_H_11_N_5_S), *m*/*z* 204.1343 (C_13_H_17_NO), *m*/*z* 218.0493 (C_9_H_7_N_5_S), and *m*/*z* 189.0773 (C_20_H_20_N_6_S). The fragment ion of *m*/*z* 204.1343 was formed by the loss of the quinazolin-4-amine moiety, and that of *m*/*z* 246.0774 was produced by cleavage between quinazolin-4-amine and the benzyl group. The fragment ion of *m*/*z* 218.0493 was formed by a neutral loss of the ethyl moiety from the ion of *m*/*z* 246.0774, and the product ion of *m*/*z* 189.0773, a doubly charged ion, was produced by the cross ring cleavage of the piperidine ring.

The major product ions in the MS^2^ spectra of yohimbine (Figure 5B) were observed at *m*/*z* 212.1248 (C_11_H_17_NO_3_) and *m*/*z* 144.0803 (C_10_H_9_N), which were formed by piperidine ring cleavage. The MS^3^ spectra of the ion of *m*/*z* 212.1248 gave ions of *m*/*z* 194.1150 (C_11_H_15_NO_2_), *m*/*z* 180.1015 (C_10_H_13_NO_2_), *m*/*z* 162.0900 (C_10_H_11_NO), *m*/*z* 152.1059 (C_9_H_13_NO), and *m*/*z* 134.0955 (C_9_H_11_N). The ion observed at *m*/*z* 194.1150 was formed by intra-molecular dehydration from the aliphatic hydroxyl (-OH) group. The ions of *m*/*z* 180.1015 and *m*/*z* 152.1059 were formed by the loss of an ester moiety, and the ions of *m*/*z* 162.0900 and *m*/*z* 134.0955 were produced by intra-molecular dehydration (loss of the aliphatic hydroxyl group) from ions of *m*/*z* 180.1015 and *m*/*z* 152.1059, respectively. These ions were confirmed by measuring their accurate mass and elemental composition. The high-resolution MS^n^ spectral library was built using all MS^n^ spectra acquired from this study; all details are provided in the Supplementary Materials (Figures S1–S15 and Table S1).

### 2.4. Analysis of Real Samples

**Figure 6:**
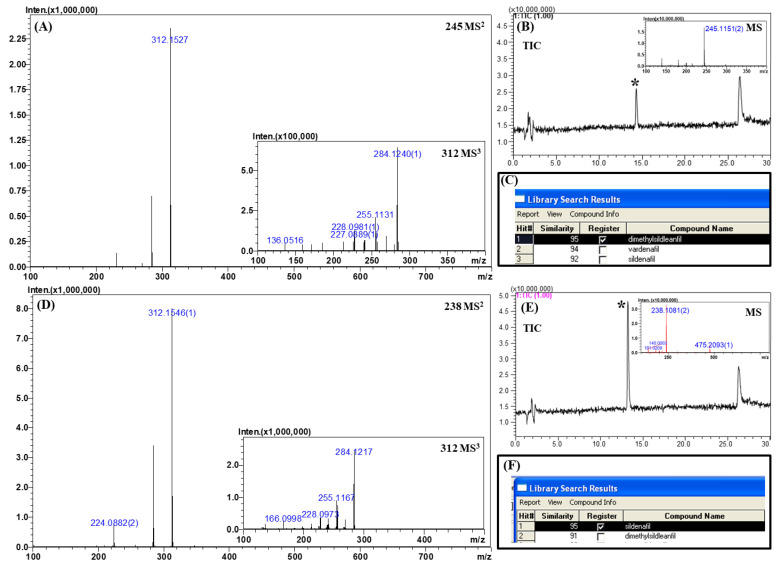
MS^n^ (*n* = 2, 3) spectra, total ion chromatograms, and library search results for real samples. (**A**) MS^2^ and MS^3^ spectra of sample 1, (**B**) chromatogram and corresponding MS spectrum of sample 1, (**C**) library search result for sample 1, (**D**) MS^2^ and MS^3^ spectra of sample 2, (**E**) chromatogram and corresponding MS spectrum of sample 2, and (**F**) library search result for sample 2.

Two positive samples (denoted as sample 1 and sample 2) were analyzed by the developed LC–MS method. The samples were extracted with methanol and diluted 1000-fold with 50% methanol. Their total ion chromatograms, MS^n^ (*n* = 1–3) spectra, and library search results are depicted in Figure 6. As shown in Figure 6, the unknown peaks were detected as the major peak even in the 1000-fold diluted samples. Two additional peaks were also observed in both samples. These peaks might be derived from the dilution solvent (50% methanol) or sample container because they appeared when the dilution solvent alone (present in the sample container) was injected into the LC–MS system (data not shown).

The unknown peak of sample 1 was observed at the retention time of 14.3 min with an accurate mass of *m*/*z* 245.1151 ([M + 2H]^2+^; Figure 6A). According to the accurate mass, the possible candidates for the unknown compound were homosildenafil, dimethylsildenafil, and vardenafil with mass errors of 10.2 ppm. On comparing the retention times of these candidates, the retention time of dimethylsildenafil was found to be similar to that of the unknown peak. Further confirmation was achieved by retrieving the MS^n^ spectral library. The library provided three matched candidates with similarity scores of over 92, namely, dimethylsildenafil, vardenafil, and sildenafil. Vardenafil was excluded from the consideration because the tandem MS spectra did not contain the diagnostic fragment ion (*m*/*z* 151.0866, C_8_H_10_N_2_O) of vardenafil. Sildenafil was also excluded because the molecular weight of the unknown was completely different from that of sildenafil. These clues collectively suggested with a high confidence level that the unknown peak belongs to dimethylsildenafil.

In the chromatogram of sample 2, the unknown peak was observed at the retention time of 13.2 min with an accurate mass of *m*/*z* 475.2093 ([M + H]^+^; Figure 6B). By considering the retention time and accurate mass, a possible candidate was sildenafil with a mass error of 6.1 ppm. The spectral library suggested two possible candidates with a similarity score of over 91, namely, sildenafil and dimethylsildenafil. As the molecular weight of the unknown peak only matched with sildenafil among the two candidates with a high confidence level, it was concluded that this unknown peak was sildenafil.

## 3. Materials and Methods

### 3.1. Chemicals and Reagents

Thirty-eight standards of PDE-5 inhibitors and their analogs were obtained from Seoul Regional Ministry of Food and Drug Safety (MFDS, Seoul, Republic of Korea). The PDE-5 inhibitors and their analogs included sildenafil, vardenafil, tadalafil, mirodenafil, udenafil, thioquinapiperifil, hydroxyvardenafil, hydroxyhongdenafil, hongdenafil, piperidinohongdenafil, dimethylsildenafil, cyclopentinafil, benzylsildenafil, thiosildenafil, dimethylthiosildenafil, chloropretadalafil, nitrodenafil, nor-neosildenafil, hydroxyhomosildenafil, acetylvardenafil, nor-neovardenafil, desulfovardenafil, aminotadalafil, yohimbine, demethylhongdenafil, oxohongdenafil, homosildenafil, xanthoanthrafil, pseudovardenafil, hydroxychlorodenafil, chlorodenafil, *N*-octyltadalafil, dichlorodenafil, carbodenafil, thiohomosildenafil, hydroxythiohomosildenafil, acetaminotadalafil, and demethyltadalafil. Phenolphthalein (internal standard, IS) was purchased from Sigma Aldrich (St. Louis, MO, USA). The detailed structures of the analytes were depicted in the Table S2. HPLC-grade methanol, acetonitrile, and water were purchased from Thermo Fisher Scientific (Fair Lawn, NJ, USA). Glacial acetic acid (99.7%), formic acid (98%), and ammonium formate were purchased from Sigma Aldrich. Stock solutions of the standards were dissolved in methanol at the appropriate concentration and stored in the refrigerator (4 °C). All standard solutions were mixed and diluted to appropriate concentration with 50% methanol.

### 3.2. Sample Preparation

Dietary supplements (100 mg) suspected of adulteration with PDE-5 inhibitor analogs were vortexed for 10 min with 10 mL of methanol and extracted in an ultrasonic bath for 30 min at room temperature.

After extraction, the samples were centrifuged at 3500 rpm for 10 min. The supernatants were filtered through a syringe filter (0.45 μm of pore size). The aliquot of the filtered supernatants was diluted with 50% methanol by a dilution factor of 100 or 1000.

### 3.3. Instrumentation and Separation Conditions

The LC–MS/MS system consisted of a Shimadzu LC-20A HPLC system and LC–IT–TOF MS equipped with an electrospray ionization source (Shimadzu, Kyoto, Japan). The chromatographic separation was achieved on the Capcell PAK C18 UG120 column (2.1 × 150 mm, 3 μm particle size, Shiseido, Tokyo, Japan) using a mobile phase comprising 0.2% acetic acid (A) and acetonitrile (B) at a flow rate of 0.2 mL/min and a temperature of 50 °C. The gradient program was as follows: 0 min, 10% B; 5 min, 20% B; 45 min, 40% B; 50 min, 85% B; 55 min, 85% B; 55.1 min, 10% B; 70 min, 10% B. The injection volume was 5 μL. The operating electrospray ionization (ESI) parameters were as follows: ion spray voltage, 4.5 kV; drying gas (N_2_), 1.5 L/min; and curved desolvation line temperature and heat block temperature, 200 °C. The MS system was calibrated prior to the analysis by using sodium trifluoroacetate solution. The HPLC and MS systems were controlled by the Lab Solution (Ver. 3.1.360) software.

### 3.4. MS^n^ Analysis

Data-dependent MS^n^ (*n* = 1–3) analysis was adopted to obtain structural information of the analytes. The precursor ion was selected over the range of *m*/*z* 100–800, and the MS^n^ spectra were obtained at the relative collision energy of 150%. Three precursor ions were selected automatically in the order of their intensity in a given spectrum (over 100,000 cps). Ion accumulation time in the ion trap was set to 10 ms for both MS and MS^n^ (*n* = 2, 3) modes. Elemental composition and mass error were calculated using accurate mass by the formula predictor software included in the Lab Solution software. The spectral libraries of the references were built on the basis of the MS^2^ and MS^3^ spectra by the library editor software included in the Lab Solution software.

### 3.5. Method Validation

Calibration curves for LC–MS were constructed with six different concentration levels from the concentration ranges listed in Table 1. The intra- and inter-day precision (CV) and accuracy (%) were estimated by analyzing five replicates at three different concentrations all within one day or over five days. The reproducibility of accurate mass measurements was estimated by analyzing six replicates.

## 4. Conclusions

An accurate screening method for PDE-5 inhibitors and their analogs in dietary supplements was developed using hybrid IT–TOF MS and validated. IT–TOF MS gave more accurate information on the structure of analytes by providing accurate masses of the parent ion as well as fragment ions, which could be converted to determine elemental composition. The spectral library of references was built against the MS^n^ spectra for retrieving the structural hits. The library suggested appropriate candidates and helped identify the unknown compounds during the real sample analysis. The combined measurement of accurate mass and retention time of the analytes facilitated accurate identification of the adulterated compounds. In conclusion, the developed LC–MS method and MS^n^ spectral library provided spectral insights for newly synthesized PDE-5 inhibitors and facilitated the rapid screening and identification of illegally adulterated analogs.

## Figures and Tables

**Table 1:** Retention times and mass errors of PDE-5 inhibitors and their analogs.

Compound	Retention Time (min)	Elemental Composition	Measured Mass	Theoretical Mass	∆*m* (mDa)	Mass Error (ppm ± SD ^a^)
Sildenafil	12.6	C_22_H_30_N_6_O_4_S	475.2125	475.2122	0.30	0.63 ± 2.21
Carbodenafil	9.3	C_24_H_32_N_6_O_3_	453.2599	453.2609	−1.02	−2.26 ± 0.54
Demethylhongdenafil	9.3	C_24_H_32_N_6_O_3_	453.2618	453.2609	0.94	2.08 ± 3.68
Hydroxyhongdenafil	9.9	C_25_H_34_N_6_O_4_	483.2728	483.2714	1.41	2.91 ± 1.70
Hongdenafil	10.6	C_25_H_34_N_6_O_3_	467.2753	467.2765	−1.19	−2.55 ± 3.09
Hydroxyhomosildenafil	10.9	C_23_H_32_N_6_O_5_S	505.2215	505.2228	−1.26	−2.49 ± 2.36
Piperidinohongdenafil	11.2	C_24_H_31_N_5_O_3_	438.2505	438.2500	0.48	1.10 ± 1.47
Homosildenafil	13.0	C_23_H_32_N_6_O_4_S	489.2278	489.2279	−0.06	−0.12 ± 0.42
Dimethylsildenafil	13.7	C_23_H_32_N_6_O_4_S	489.2291	489.2279	1.24	2.54 ± 0.95
Udenafil	15.0	C_25_H_36_N_6_O_4_S	517.2567	517.2592	−2.52	−4.48 ± 0.63
Cyclopentinafil	16.2	C_26_H_36_N_6_O_4_S	529.2591	529.2592	−0.09	−0.17 ± 0.52
Thiosildenafil	23.6	C_22_H_30_N_6_O_3_S_2_	491.1882	491.1893	−1.09	−2.22 ± 0.94
Hydroxythiohomosildenafil	23.7	C_23_H_32_N_6_O_4_S_2_	521.1956	521.1999	−4.32	−8.30 ± 2.15
Thiohomosildenafil	24.4	C_23_H_32_N_6_O_3_S_2_	505.2013	505.2050	−3.73	−7.37 ± 2.56
Dimethylthiosildenafil	25.1	C_23_H_32_N_6_O_3_S_2_	505.2018	505.2050	−3.23	−6.38 ± 2.42
Oxohongdenafil	28.9	C_25_H_32_N_6_O_4_	481.2569	481.2558	1.11	2.30 ± 1.22
Benzylsildenafil	30.6	C_28_H_34_N_6_O_4_S	551.2403	551.2435	−3.19	−5.79 ± 1.20
Hydroxychlorodenafil	40.5	C_19_H_23_N_4_O_3_Cl	391.1528	391.1531	−0.28	−0.72 ± 1.60
Chlorodenafil	48.4	C_19_H_21_N_4_O_3_Cl	389.1377	389.1375	0.17	0.43 ± 1.10
Nitrodenafil	49.6	C_17_H_19_N_5_O_4_	358.1512	358.1510	0.17	0.47 ± 1.59
Nor-neosildenafil	51.7	C_22_H_29_N_5_O_4_S	460.2014	460.2013	0.08	0.18 ± 1.72
Dichlorodenafil	53.6	C_19_H_20_N_4_O_2_Cl_2_	407.1038	407.1036	0.20	0.49 ± 1.81
Vardenafil	11.1	C_23_H_32_N_6_O_4_S	489.2296	489.2279	1.67	3.42 ± 0.86
Acetylvardenafil	9.3	C_25_H_34_N_6_O_3_	467.2758	467.2765	−0.69	−1.48 ± 5.47
Hydroxyvardenafil	10.9	C_23_H_32_N_6_O_5_S	505.2209	505.2228	−1.86	−3.68 ± 0.84
Nor-neovardenafil	23.9	C_18_H_20_N_4_O_4_	357.1557	357.1557	−0.02	−0.05 ± 0.86
Desulfovardenafil	37.1	C_17_H_20_N_4_O_2_	313.1651	313.1659	−0.80	−2.55 ± 0.88
Pseudovardenafil	48.5	C_22_H_29_N_5_O_4_S	460.2013	460.2013	−0.05	−0.11 ± 0.64
Tadalafil	29.8	C_22_H_19_N_3_O_4_	390.1449	390.1448	0.10	0.26 ± 1.42
Aminotadalafil	24.4	C_21_H_18_N_4_O_4_	391.1410	391.1401	0.88	2.26 ± 1.57
Acetaminotadalafil	24.6	C_23_H_20_N_4_O_5_	433.1502	433.1506	−0.42	−0.96 ± 1.81
Demethyltadalafil	25.5	C_21_H_17_N_3_O_4_	376.1293	376.1292	0.07	0.18 ± 1.36
Chloropretadalafil	48.5	C_22_H_19_N_2_O_5_Cl	427.1031	427.1055	−2.42	−5.66 ± 1.62
*N*-Octyltadalafil	53.1	C_29_H_33_N_3_O_4_	488.2559	488.2544	1.48	3.04 ± 3.23
Yohimbine	6.4	C_21_H_26_N_2_O_3_	532.2580	532.2588	−0.82	−1.55 ± 0.97
Mirodenafil	22.4	C_26_H_37_N_5_O_5_S	355.2012	355.2016	−0.43	−1.22 ± 1.73
Thioquinapiperifil	10.8	C_24_H_28_N_6_OS	449.2098	449.2118	−1.96	−4.36 ± 2.06
Xanthoanthrafil	30.2	C_19_H_23_N_3_O_6_	390.1660	390.1660	−0.02	−0.04 ± 1.94
IS ^b^ (Phenolphthalein)	27.7	C_20_H_14_O_4_	319.0934	319.0965	−3.10	9.72

^a^ Standard deviation; ^b^ Internal standard.

**Table 2:** Linear regression equations and linear correlation coefficients of PDE-5 inhibitors and their analogs.

Compound	Equation	Range (μg/mL)	*R* ^2^
Sildenafil	*y* = 0.169 *x* + 0.006	0.30–0.80	0.993
Carbodenafil	*y* = 0.430 *x* − 0.008	0.16–0.43	0.997
Hongdenafil	*y* = 0.783 *x* + 0.037	0.38–1.00	0.997
Hydroxyhongdenafil	*y* = 0.506 *x* + 0.170	0.30–0.80	0.994
Demethylhongdenafil	*y* = 0.403 *x* + 0.020	0.32–0.85	0.996
Piperidinohongdenafil	*y* = 0.504 *x* + 0.052	0.28–0.74	0.996
Hydroxyhomosildenafil	*y* = 0.490 *x* + 0.074	0.74–1.98	0.994
Homosildenafil	*y* = 0.321 *x* + 0.025	0.20–0.55	0.998
Dimethylsildenafil	*y* = 0.409 *x* + 0.030	0.30–0.80	0.995
Udenafil	*y* = 0.504 *x* + 0.133	0.30–0.80	0.999
Cyclopentinafil	*y* = 0.389 *x* + 0.040	0.30–0.80	0.997
Thiosildenafil	*y* = 0.165 *x* + 0.026	1.20–3.20	0.996
Hydroxythiohomosildenafil	*y* = 0.198 *x* + 0.003	0.75–2.00	0.998
Thiohomosildenafil	*y* = 0.252 *x* + 0.007	0.15–0.40	0.993
Dimethylthiosildenafil	*y* = 0.255 *x* − 0.010	0.18–0.47	0.995
Oxohongdenafil	*y* = 0.237 *x* + 0.034	0.60–1.60	0.993
Benzylsildenafil	*y* = 0.157 *x* + 0.010	0.31–0.84	0.993
Hydroxychlorodenafil	*y* = 0.302 *x* + 0.011	0.25–0.66	0.998
Chlorodenafil	*y* = 0.341 *x* + 0.386	1.50–4.00	0.990
Nitrodenafil	*y* = 0.444 *x* + 0.016	0.15–0.40	0.994
Nor-neosildenafil	*y* = 0.356 *x* + 0.020	0.20–0.54	0.993
Dichlorodenafil	*y* = 0.107 *x* + 0.017	0.38–1.00	0.992
Vardenafil	*y* = 0.381 *x* + 0.014	0.15–0.40	0.999
Acetylvardenafil	*y* = 0.501 *x* + 0.046	0.31–0.83	0.992
Hydroxyvardenafil	*y* = 0.610 *x* + 0.055	0.75–2.00	0.998
Nor-neovardenafil	*y* = 0.729 *x* + 0.027	0.17–0.45	0.993
Desulfovardenafil	*y* = 1.289 *x* + 0.052	0.15–0.40	0.991
Pseudovardenafil	*y* = 0.912 *x* + 0.045	0.15–0.40	0.992
Tadalafil	*y* = 0.090 *x* + 0.015	0.70–1.87	0.997
Aminotadalafil	*y* = 0.048 *x* + 0.018	1.20–3.20	0.997
Acetaminotadalafil	*y* = 0.108 *x* + 0.022	0.38–1.00	0.990
Demethyltadalafil	*y* = 0.088 *x* + 0.015	0.75–2.00	0.993
Chloropretadalafil	*y* = 0.027 *x* + 0.044	1.20–3.20	0.992
*N*-Octyltadalafil	*y* = 0.043 *x* + 0.019	0.75–2.00	0.996
Yohimbine	*y* = 0.539 *x* + 0.028	0.13–0.34	0.995
Mirodenafil	*y* = 0.939 *x* + 0.004	0.17–0.45	0.991
Thioquinapiperifil	*y* = 0.198 *x* − 0.016	0.15–0.40	0.994
Xanthoanthrafil	*y* = 0.176 *x* + 0.020	0.60–1.60	0.992

**Table 3:** Intra- and inter-day accuracy and precision of PDE-5 inhibitors and their analogs.

Compound	Intra-Day (*n* = 5)						Inter-Day (*n* = 5)					
	Accuracy (%)			Precision (CV %)			Accuracy (%)			Precision (CV %)		
	L ^a^	M ^b^	H ^c^	L	M	H	L	M	H	L	M	H
Sildenafil	104.2	103.7	101.6	5.5	3.1	3.9	99.6	103.4	100.1	2.8	3.6	1.9
Carbodenafil	103.7	101.6	99.7	4.8	2.5	5.2	100.9	101.7	96.8	4.4	5.1	4.8
Hongdenafil	106.0	103.8	99.2	4.0	3.4	3.1	97.1	103.2	98.9	5.9	4.3	4.1
Hydroxyhongdenafil	101.2	98.6	97.6	4.2	2.2	2.0	94.1	98.4	98.2	5.3	3.3	2.0
Demethylhongdenafil	102.4	99.6	98.7	5.2	2.3	5.5	94.4	99.5	96.9	4.2	3.9	2.5
Piperidinohongdenafil	104.0	103.4	96.7	7.1	2.8	2.8	93.0	102.0	97.6	5.3	4.4	1.8
Hydroxyhomosildenafil	107.1	104.7	101.8	6.7	0.7	3.4	94.5	99.9	98.3	5.3	5.3	3.5
Homosildenafil	102.5	103.6	102.1	4.3	3.1	2.9	94.5	105.1	97.8	3.7	2.1	3.3
Dimethylsildenafil	105.6	104.7	102.0	7.8	1.1	1.7	96.0	101.1	100.0	1.2	3.3	3.3
Udenafil	98.9	104.7	104.7	3.3	3.0	1.4	93.8	103.6	100.7	6.5	2.2	6.1
Cyclopentinafil	100.2	100.9	102.2	4.1	1.5	2.3	95.4	102.7	98.7	5.4	6.1	3.2
Thiosildenafil	105.3	105.2	104.3	7.5	2.8	2.7	93.3	100.6	102.2	4.9	7.3	2.0
Hydroxythiohomosildenafil	102.9	104.2	102.5	5.2	2.5	2.7	97.1	104.4	101.9	4.6	1.6	5.4
Thiohomosildenafil	103.9	103.7	101.4	9.2	4.0	4.0	96.0	99.0	97.8	7.9	4.9	6.3
Dimethylthiosildenafil	99.5	96.5	99.9	3.7	6.7	7.6	96.2	101.2	97.0	8.4	6.8	6.1
Oxohongdenafil	98.0	101.8	99.9	2.3	2.1	2.4	91.3	100.4	99.6	4.0	3.4	1.4
Benzylsildenafil	96.2	96.6	99.3	3.1	3.5	5.6	91.8	99.7	97.7	4.3	3.4	3.2
Hydroxychlorodenafil	92.5	98.1	100.1	1.4	2.2	3.1	91.1	99.1	97.3	3.1	3.7	2.7
Chlorodenafil	97.0	103.3	102.2	4.6	4.5	1.9	89.7	103.0	97.8	7.8	4.2	3.4
Nitrodenafil	98.3	99.8	102.9	3.7	3.0	4.2	89.7	101.0	100.6	9.3	5.7	3.3
Nor-neosildenafil	105.1	105.3	100.0	6.5	1.4	2.9	92.3	102.3	97.4	3.0	3.2	1.9
Dichlorodenafil	93.5	100.7	101.5	2.6	6.2	4.8	90.9	103.4	97.8	4.1	6.3	3.4
Vardenafil	100.8	98.7	96.4	4.4	3.0	3.7	93.0	100.4	95.8	4.7	4.4	2.9
Acetylvardenafil	102.1	103.0	99.7	5.0	4.7	3.5	96.0	103.6	101.0	4.1	2.9	5.2
Hydroxyvardenafil	104.9	102.6	98.7	5.8	1.9	3.1	96.6	100.6	97.9	5.9	5.3	2.1
Nor-neovardenafil	87.8	96.6	98.1	5.9	3.6	1.3	97.0	103.1	97.7	8.7	5.9	1.6
Desulfovardenafil	86.7	94.7	96.5	5.2	3.3	2.6	91.1	100.9	97.4	3.2	5.4	1.8
Pseudovardenafil	101.0	101.2	100.5	3.0	1.2	2.8	91.3	101.0	98.5	7.3	4.1	3.2
Tadalafil	94.2	94.3	95.7	3.1	2.5	3.4	91.9	96.6	99.1	7.9	5.6	3.4
Aminotadalafil	98.4	97.7	98.7	3.2	3.9	4.7	92.6	102.3	99.9	5.4	3.5	1.3
Acetaminotadalafil	99.2	100.2	101.3	4.6	6.6	4.2	90.3	99.4	100.3	7.2	5.7	3.2
Demethyltadalafil	92.1	100.7	102.4	4.5	2.4	2.6	89.8	102.7	101.0	5.1	2.0	3.5
Chloropretadalafil	106.7	105.6	105.0	2.6	2.2	3.5	98.0	105.6	105.7	10.5	2.9	2.1
*N*-Octyltadalafil	107.8	106.0	98.1	7.9	0.6	4.8	95.4	103.9	97.9	6.5	5.7	1.8
Yohimbine	106.1	104.0	101.3	5.6	3.4	3.6	92.6	101.2	97.0	6.3	5.2	2.1
Mirodenafil	107.1	100.2	95.9	5.1	1.3	3.2	96.2	101.2	95.8	5.2	4.1	4.4
Thioquinapiperifil	112.0	105.0	104.4	5.1	2.9	1.9	104.4	99.3	100.0	3.3	7.5	3.6
Xanthoanthrafil	91.8	99.5	102.3	3.0	2.1	4.6	92.6	99.3	98.4	4.9	4.4	2.1

^a^ L: The lowest concentration in the calibration curve; ^b^ M: Medium concentration in the calibration curve; ^c^ H: The highest concentration in the calibration curve.

**Table 4:** Accurate masses and mass errors for fragment and precursor ions of PDE-5 inhibitors and their analogs.

Compound	Elemental Composition	Measured Mass	Theoretical Mass	∆*m* (mDa)	Error (ppm)
Sildenafil	C_22_H_30_N_6_O_4_S ^a^	238.1087 ^b^	238.1100 ^b^	−1.3	−5.46
	C_17_H_20_N_4_O_4_S	377.1263	377.1278	−1.5	−3.98
	C_17_H_19_N_4_O_2_	312.1573	312.1581	−0.8	−2.56
	C_15_H_15_N_4_O_2_	284.1221	284.1268	−4.7	−16.54
Thiosildenafil	C_22_H_30_N_6_O_3_S_2_^a^	246.0977 ^b^	246.0983 ^b^	−0.6	−2.50
	C_17_H_19_N_4_OS	328.1344	328.1352	−0.8	−2.44
	C_15_H_15_N_4_OS	300.1022	300.1039	−1.7	−5.66
Vardenafil	C_23_H_32_N_6_O_4_S ^a^	245.1168 ^b^	245.1176 ^b^	−0.8	−3.26
	C_17_H_19_N_4_O_2_	312.1555	312.1581	−2.6	−8.33
	C_15_H_15_N_4_O_2_	284.1253	284.1268	−1.5	−5.28
	C_8_H_10_N_2_O	151.0877	151.0866	1.1	7.28
Tadalafil	C_22_H_19_N_3_O_4_^a^	390.1454	390.1448	0.6	1.54
	C_15_H_13_N_3_O_2_	268.1050	268.1081	−3.1	−11.56
	C_17_H_11_NO_2_	262.0866	262.0863	0.3	1.14
	C_14_H_13_N_3_O	240.1099	240.1131	−3.2	−13.33
	C_12_H_8_N_2_O	197.0707	197.0709	−0.2	−1.01
	C_11_H_8_N_2_	169.0736	169.0760	−2.4	−14.19
	C_8_H_6_O_2_	135.0451	135.0441	1.0	7.40
Mirodenafil	C_26_H_37_N_5_O_5_S ^a^	266.6326 ^b^	266.6330 ^b^	−0.4	−1.50
	C_20_H_24_N_3_O_2_	339.1914	339.1941	−2.7	−7.96
	C_17_H_18_N_3_O_2_	297.1444	297.1472	−2.8	−9.42
	C_23_H_31_N_5_O_5_S	245.6061 ^b^	245.6096 ^b^	−3.5	−14.25
	C_23_H_29_N_5_O_4_S	236.6006 ^b^	236.6043 ^b^	−3.7	−15.64
Xanthoanthrafil	C_19_H_23_N_3_O_6_	390.1660	390.1660	0.0	0.00
	C_9_H_10_O_2_	151.0740	151.0754	−1.4	18.17
Thioquinapiperifil	C_24_H_28_N_6_OS ^a^	225.1093 ^b^	225.1095 ^b^	−0.2	−0.89
	C_11_H_11_N_5_S	246.0774	246.0808	−3.4	−13.82
	C_9_H_7_N_5_S	218.0493	218.0495	−0.2	−0.92
	C_13_H_17_NO	204.1343	204.1383	−4.0	−19.59
Yohimbine	C_21_H_26_N_2_O_3_^a^	355.2017	355.2016	0.1	0.28
	C_11_H_17_NO_3_	212.1248	212.1281	−3.3	−15.56
	C_11_H_15_NO_2_	194.1150	194.1176	−2.6	−13.39
	C_10_H_9_N	144.0803	144.0808	−0.5	−3.47
	C_10_H_13_NO_2_	180.1015	180.1019	−0.4	−2.22
	C_10_H_11_NO	162.0900	162.0913	−1.3	−8.02
	C_9_H_11_N	134.0955	134.0964	−0.9	−6.71

^a^ Precursor ion; ^b^ Multiply charged protonated ion [M + 2H]^2+^_._

## Supplementary Materials

The following are available online, Figure S1: Representative MS^n^ (*n* = 2, 3) spectra and proposed fragmentation mechanisms of homosildenafil (**A**) and dimethylsildenafil (**B**). The bracketed numbers next to the *m*/*z* values indicate the charge state of the ions; Figure S2: Representative MS^n^ (*n* = 2, 3) spectra and proposed fragmentation mechanisms of thiohomosildenafil (**A**) and dimethylthiosildenafil (**B**). The bracketed numbers next to the *m*/*z* values indicate the charge state of the ions; Figure S3: Representative MS^n^ (*n* = 2, 3) spectra and proposed fragmentation mechanisms of benzylsildenafil (**A**) and carbodenafil (**B**). The bracketed numbers next to the *m*/*z* values indicate the charge state of the ions; Figure S4: Representative MS^n^ (*n* = 2, 3) spectra and proposed fragmentation mechanisms of nor-neosildenafil (**A**) and cyclopentinafil (**B**). The bracketed numbers next to the *m*/*z* values indicate the charge state of the ions; Figure S5: Representative MS^2^ spectra and proposed fragmentation mechanisms of hydroxythiohomosildenafil (**A**) and hydroxyhomosildenafil (**B**). The bracketed numbers next to the *m*/*z* values indicate the charge state of the ions; Figure S6: Representative MS^n^ (*n* = 2, 3) spectra and proposed fragmentation mechanisms of chlorodenafil (**A**) and dichlorodenafil (**B**). The bracketed numbers next to the *m*/*z* values indicate the charge state of the ions; Figure S7: Representative MS^2^ spectra and proposed fragmentation mechanisms of hydroxychlorodenafil (**A**) and nitrodenafil (**B**). The bracketed numbers next to the *m*/*z* values indicate the charge state of the ions; Figure S8: Representative MS^n^ (*n* = 2, 3) spectra and proposed fragmentation mechanisms of udenafil (**A**) and hongdenafil (**B**). The bracketed numbers next to the *m*/*z* values indicate the charge state of the ions; Figure S9: Representative MS^2^ and proposed fragmentation mechanisms of piperidinohongdenafil (**A**) and demethylhongdenafil (**B**). The bracketed numbers next to the *m*/*z* values indicate the charge state of the ions; Figure S10: Representative MS^2^ spectra and proposed fragmentation mechanisms of hydroxyhongdenafil (**A**) and oxohongdenafil (**B**). The bracketed numbers next to the *m*/*z* values indicate the charge state of the ions; Figure S11: Representative MS^2^ spectra and proposed fragmentation mechanisms of desolfovardenafil (**A**) and nor-neovardenafil (**B**). The bracketed numbers next to the *m*/*z* values indicate the charge state of the ions; Figure S12: Representative MS^2^ spectra and proposed fragmentation mechanisms of pseudovardenafil (**A**) and acetylvardenafil (**B**). The bracketed numbers next to the *m*/*z* values indicate the charge state of the ions; Figure S13: Representative MS^2^ spectra and proposed fragmentation mechanisms of hydroxyvardenafil (**A**) and acetaminotadalafil (**B**). The bracketed numbers next to the *m*/*z* values indicate the charge state of the ions; Figure S14: Representative MS^2^ spectra and proposed fragmentation mechanisms of aminotadalafil (**A**) and demethyltadalafil (**B**). The bracketed numbers next to the *m*/*z* values indicate the charge state of the ions; Figure S15: Representative MS^n^ (*n* = 2, 3) spectra and proposed fragmentation mechanisms of chloropretadalafil (**A**) and *N*-octyltadalafil (**B**). The bracketed numbers next to the *m*/*z* values indicate the charge state of the ions; Table S1: Accurate masses and mass errors for fragment and precursor ions of PDE-5 inhibitors and their analogs; Table S2: The structures of PDE-5 inhibitors and their analogues.
