# Supplementary material for: Screening of Phosphodiesterase-5 Inhibitors and Their Analogs in Dietary Supplements by Liquid Chromatography–Hybrid Ion Trap–Time of Flight Mass Spectrometry
Source: Molecules. 2020 Jun 12;25(12):2734. doi: 10.3390/molecules25122734 (PMC7355528; doi:10.3390/molecules25122734)
Supplement: Supplementary file 1 [file molecules-25-02734-s001.pdf]

**Figure S1.** Representative MS$^n$ ($n$ = 2, 3) spectra and proposed fragmentation mechanisms of

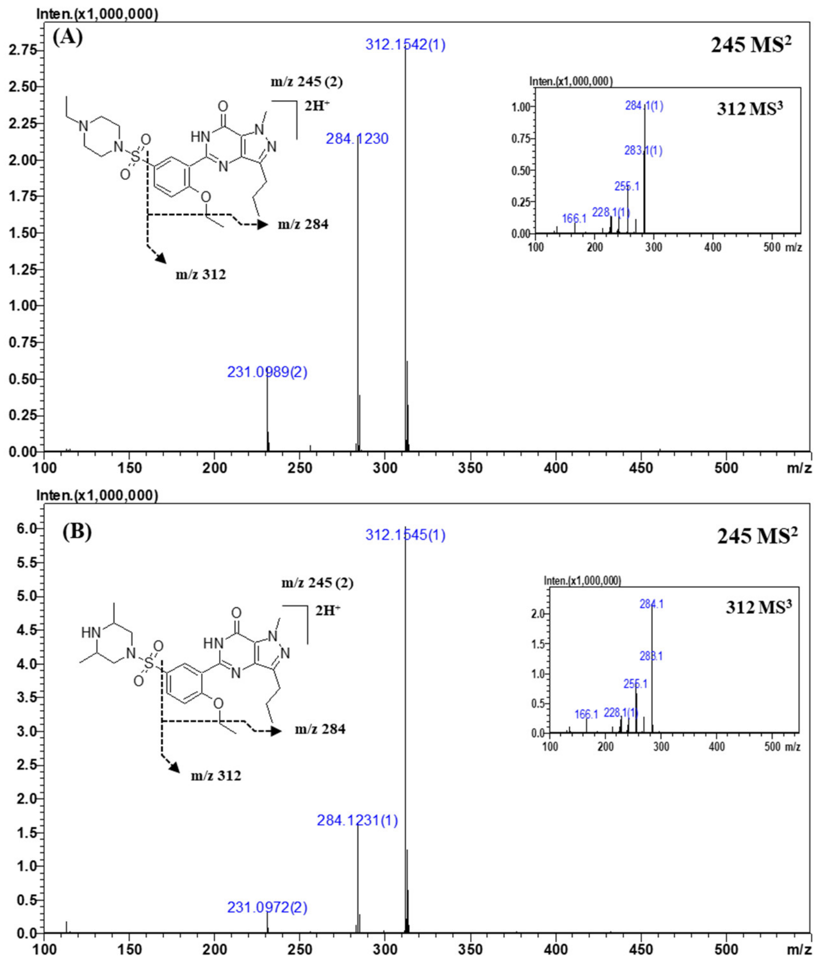


homosildenafil (A) and dimethylsildenafil (B). The bracketed numbers next to the *m*/*z* values indicate the charge state of the ions.

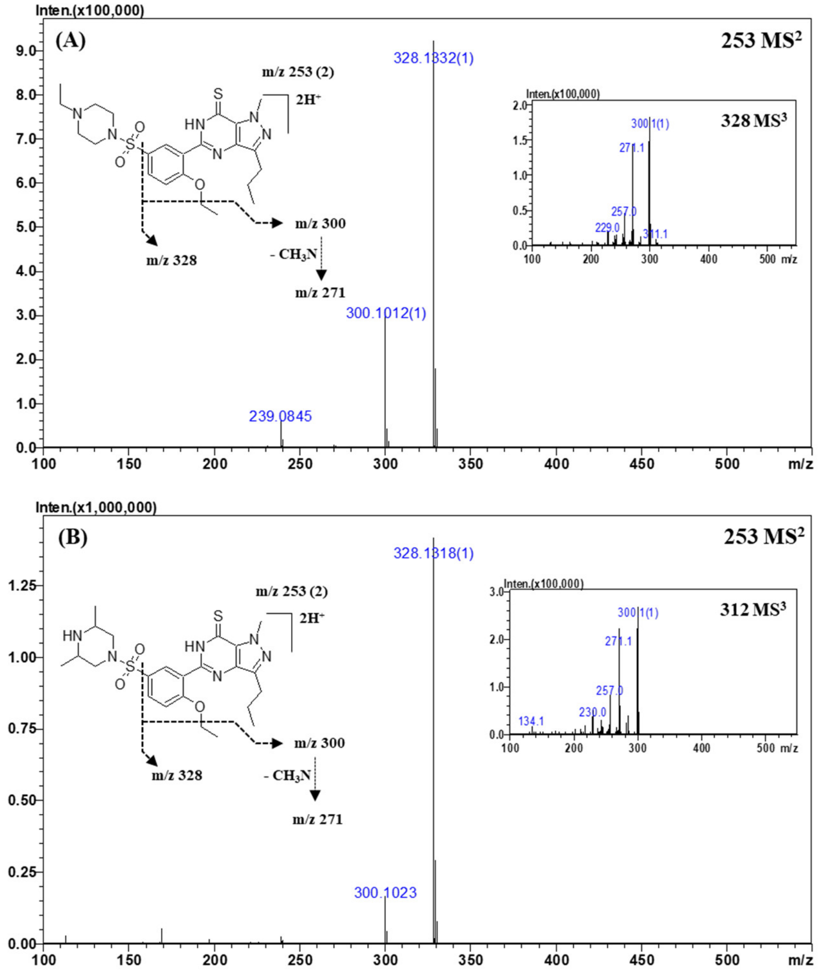


**Figure S2.** Representative MS$^n$ ($n$ = 2, 3) spectra and proposed fragmentation mechanisms of thiohomosildenafil (A) and dimethylthiosildenafil (B). The bracketed numbers next to the *m*/*z* values indicate the charge state of the ions.

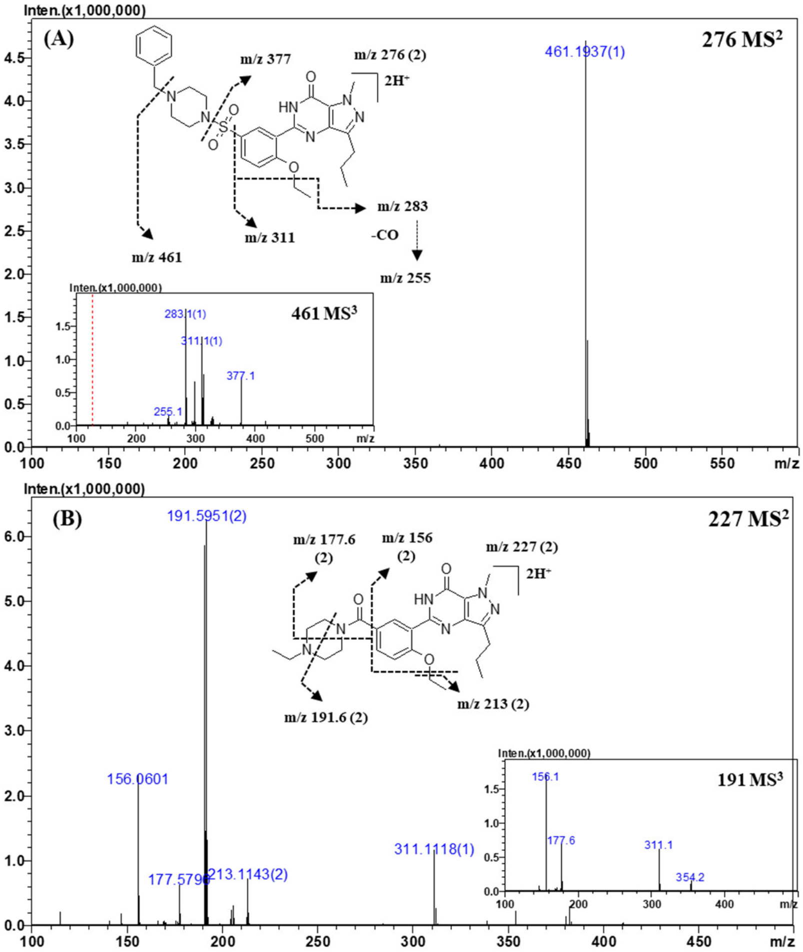


**Figure S3.** Representative $MS^n$ ($n$ = 2, 3) spectra and proposed fragmentation mechanisms of benzylsildenafil (A) and carbodenafil (B). The bracketed numbers next to the *m/z* values indicate the charge state of the ions.

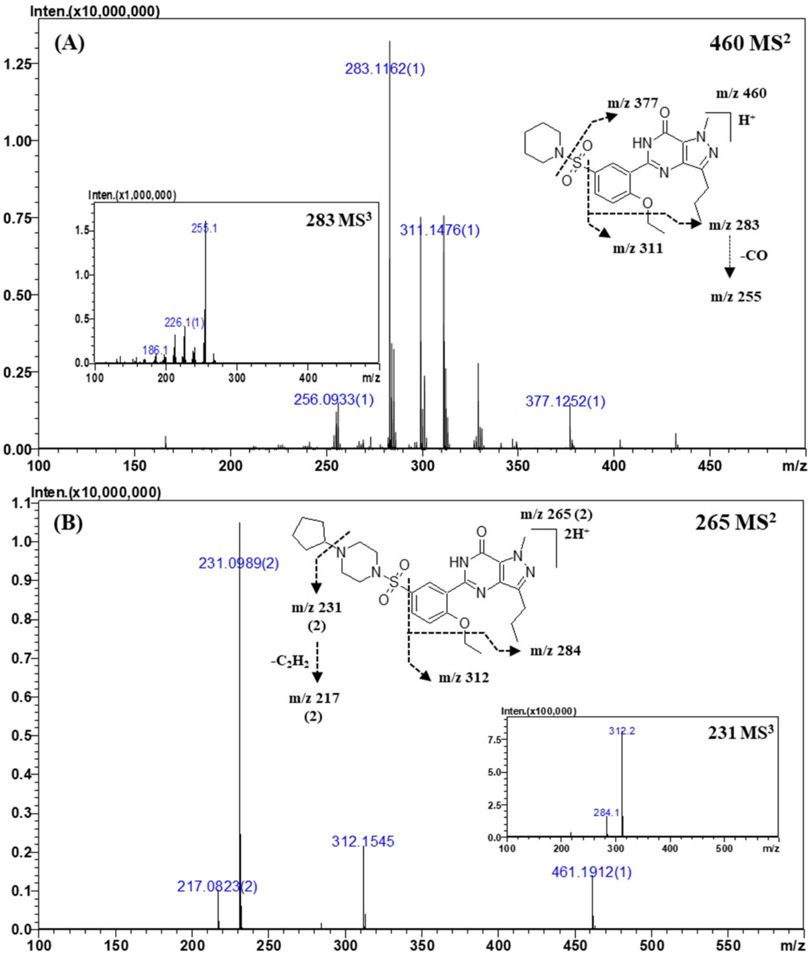


**Figure S4.** Representative MS$^n$ ($n$ = 2, 3) spectra and proposed fragmentation mechanisms of nor-neosildenafil (A) and cyclopentinafil (B). The bracketed numbers next to the *m*/*z* values indicate the charge state of the ions.

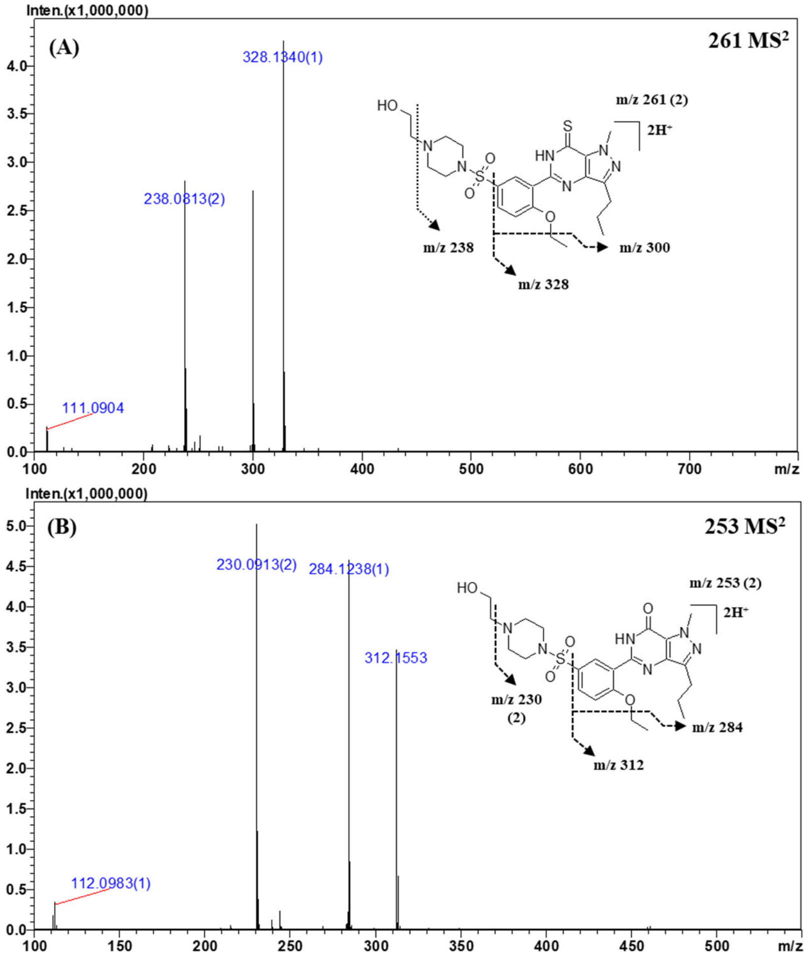


**Figure S5.** Representative $MS^2$ spectra and proposed fragmentation mechanisms of hydroxythiohomosildenafil (A) and hydroxyhomosildenafil (B). The bracketed numbers next to the *m*/*z* values indicate the charge state of the ions.

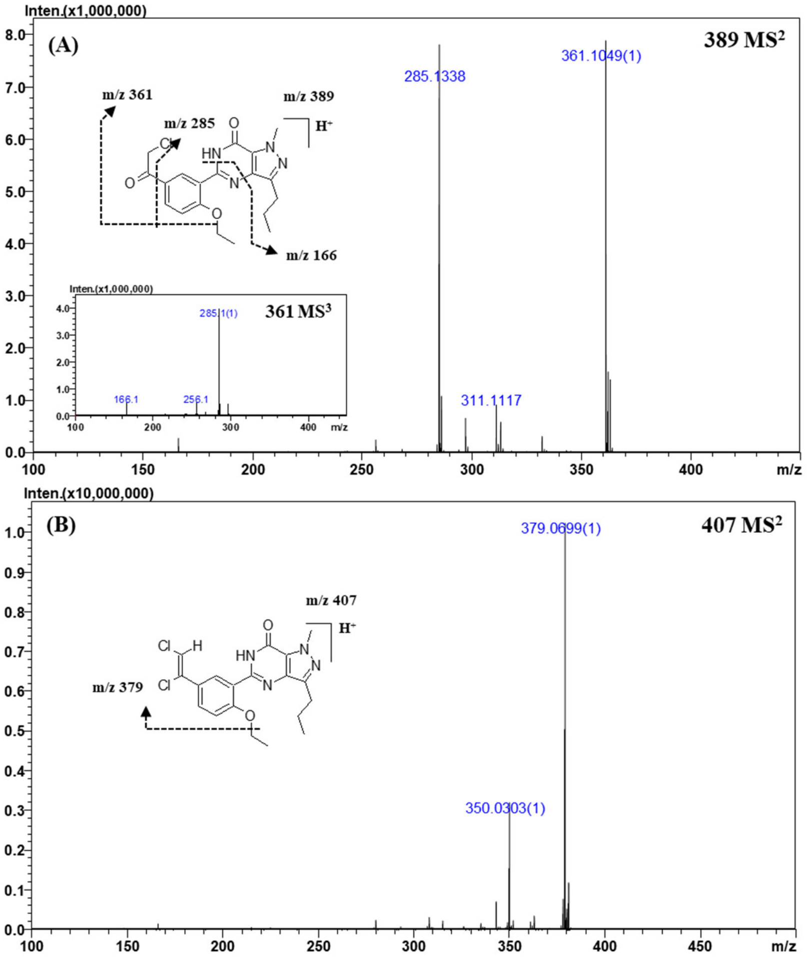


**Figure S6.** Representative MS$^n$ ($n$ = 2, 3) spectra and proposed fragmentation mechanisms of chlorodenafil (A) and dichlorodenafil (B). The bracketed numbers next to the *m*/*z* values indicate the charge state of the ions.

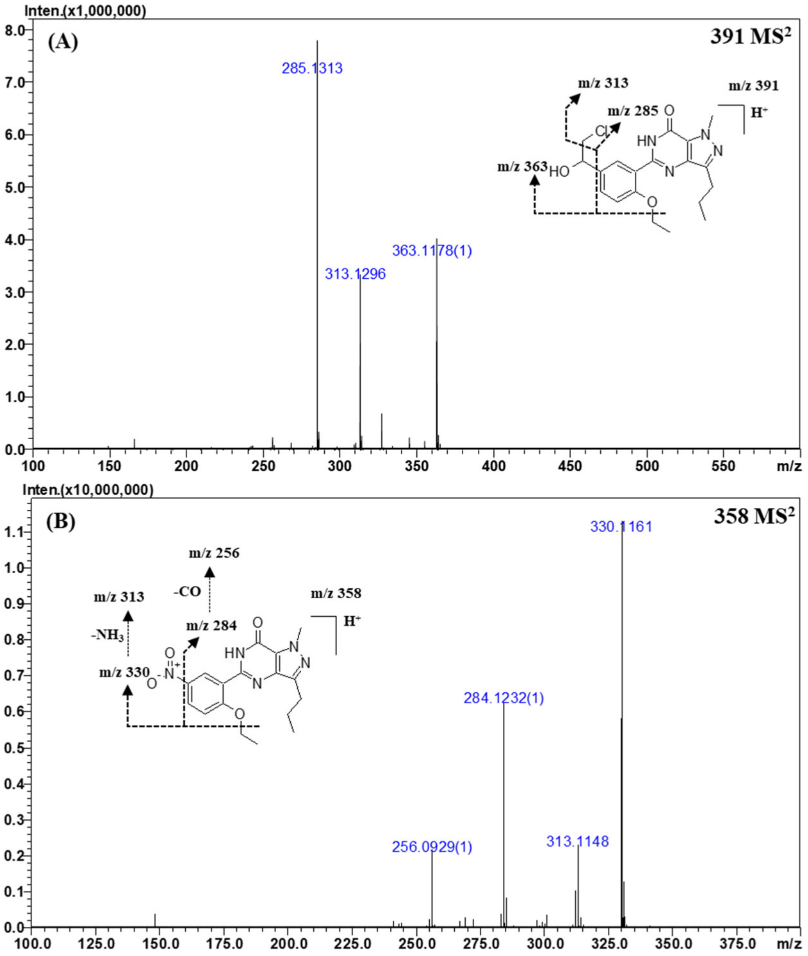


**Figure S7.** Representative MS² spectra and proposed fragmentation mechanisms of hydroxychlorodenafil (A) and nitrodenafil (B). The bracketed numbers next to the *m*/*z* values indicate the charge state of the ions.

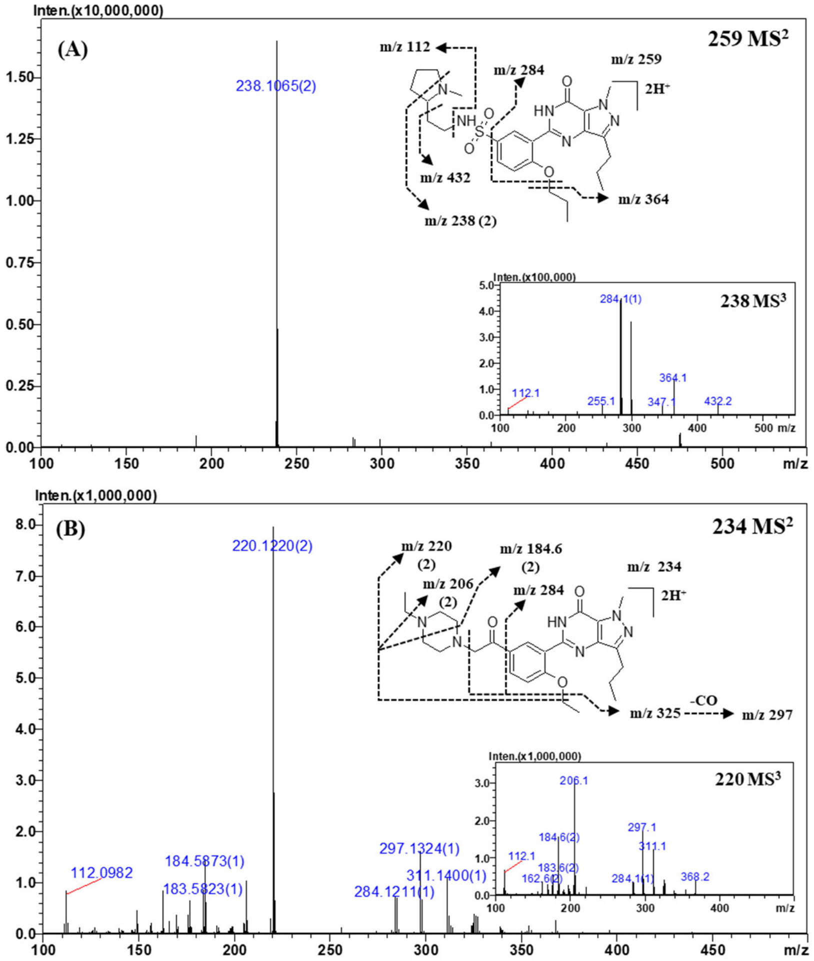


**Figure S8.** Representative MS$^n$ ($n$ = 2, 3) spectra and proposed fragmentation mechanisms of udenafil (A) and hongdenafil (B). The bracketed numbers next to the *m/z* values indicate the charge state of the ions.

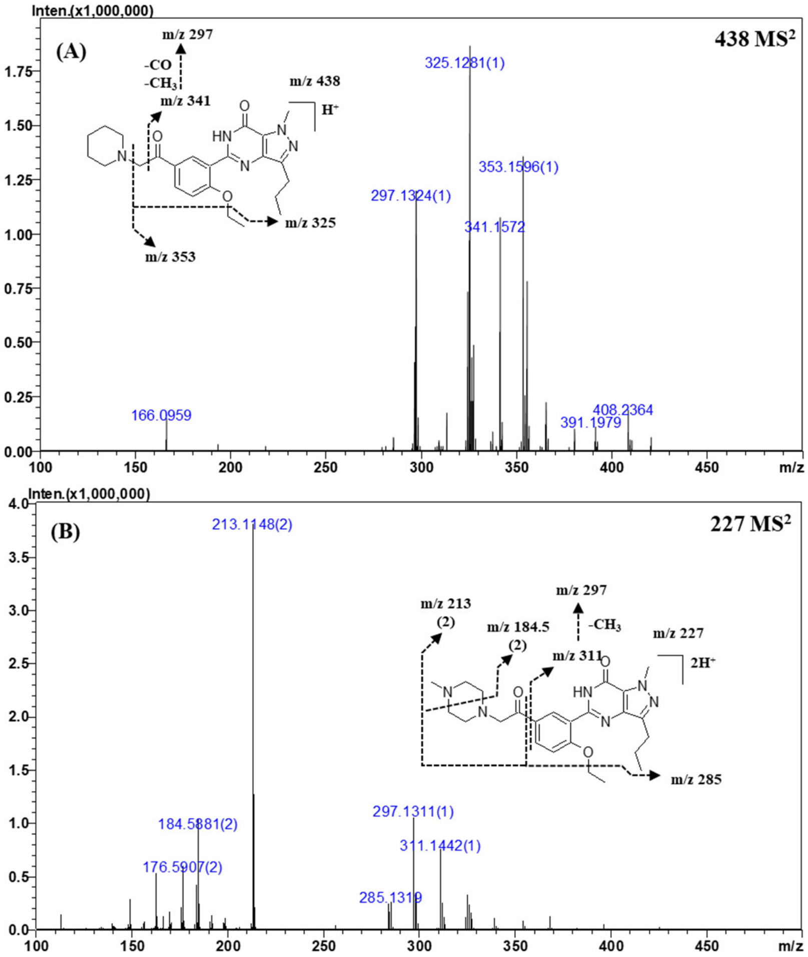


**Figure S9.** Representative MS² and proposed fragmentation mechanisms of piperidinohongdenafil (A) and demethylhongdenafil (B). The bracketed numbers next to the *m*/*z* values indicate the charge state of the ions.

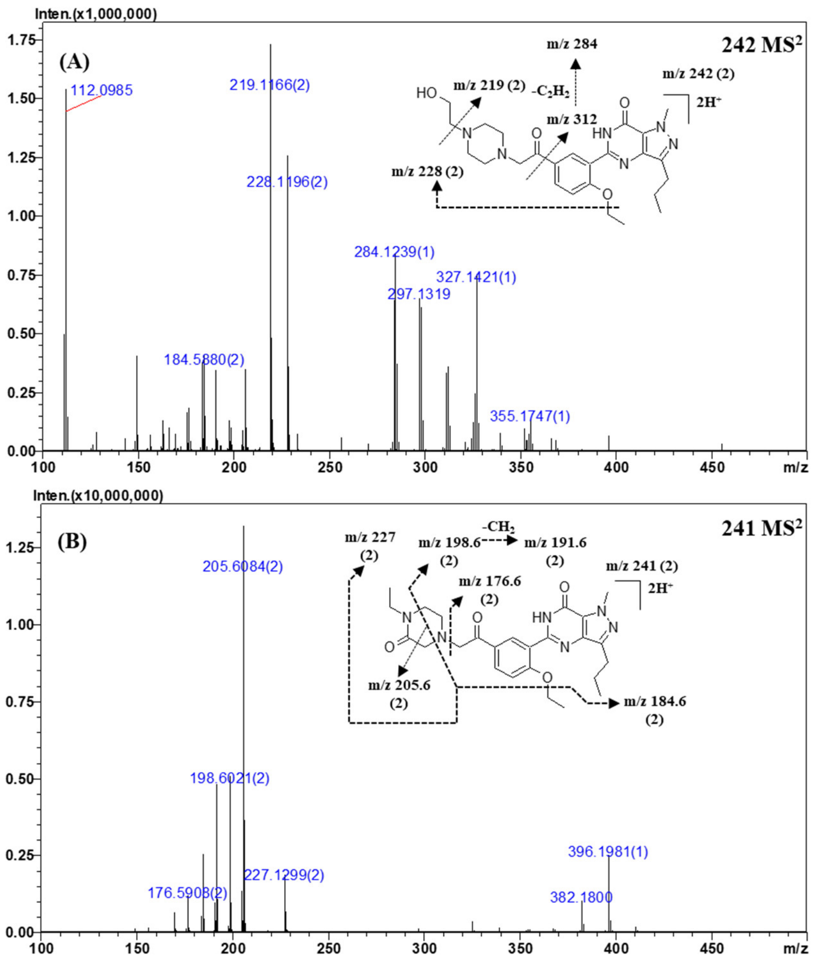


**Figure S10.** Representative MS² spectra and proposed fragmentation mechanisms of hydroxyhongdenafil (A) and oxohongdenafil (B). The bracketed numbers next to the *m*/*z* values indicate the charge state of the ions.

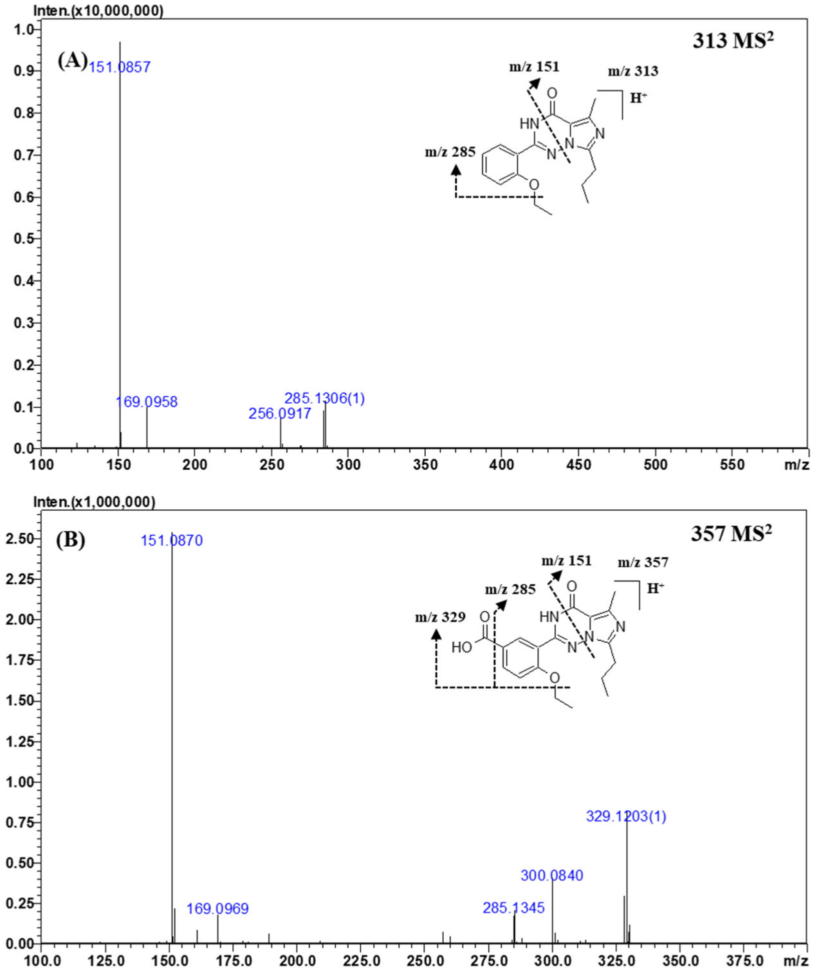


**Figure S11.** Representative MS² spectra and proposed fragmentation mechanisms of desolfovardenafil (A) and nor-neovardenafil (B). The bracketed numbers next to the *m*/*z* values indicate the charge state of the ions.

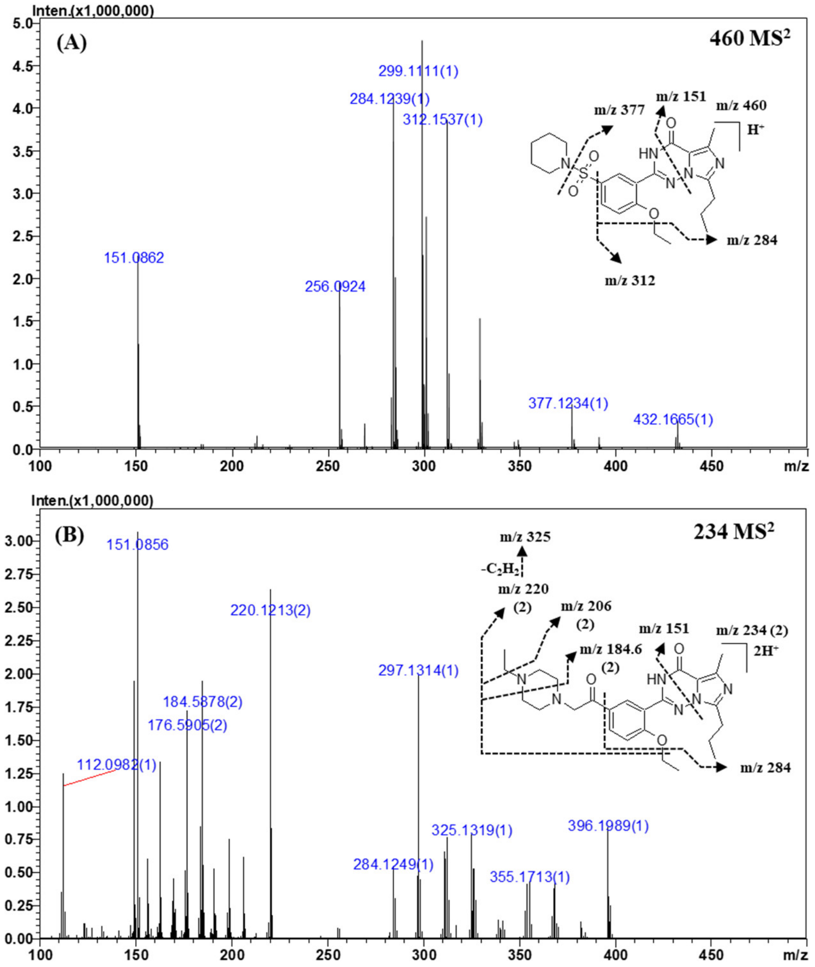


**Figure S12.** Representative $MS^2$ spectra and proposed fragmentation mechanisms of pseudovardenafil (A) and acetylvardenafil (B). The bracketed numbers next to the *m*/*z* values indicate the charge state of the ions.

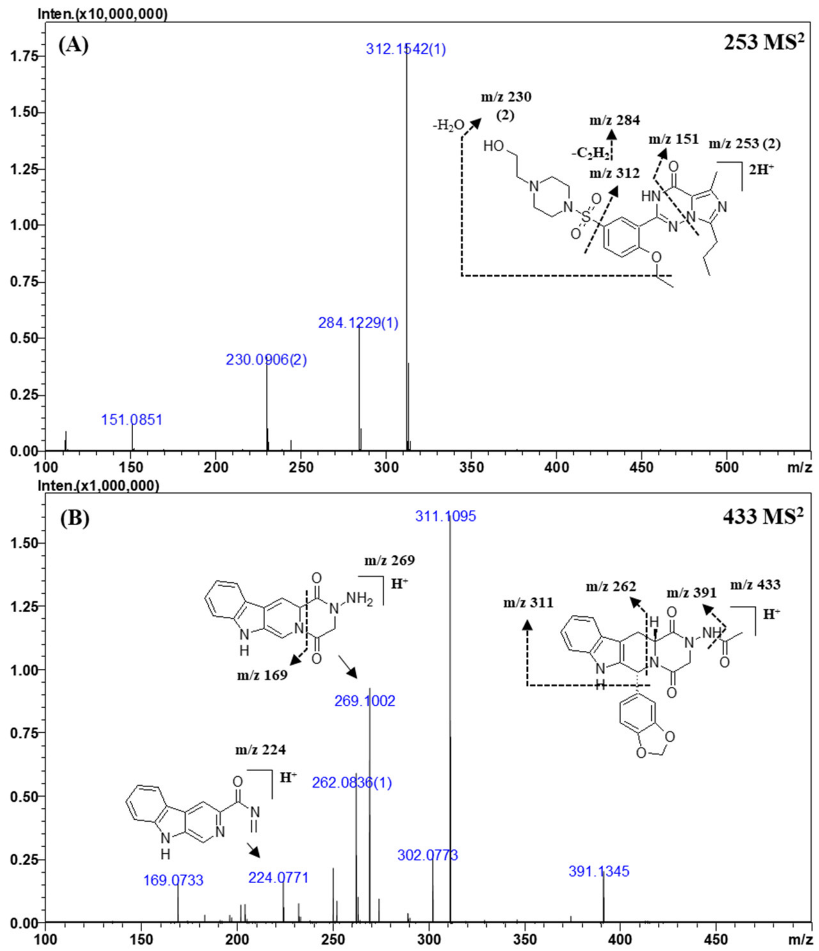


**Figure S13.** Representative MS² spectra and proposed fragmentation mechanisms of hydroxyvardenafil (A) and acetaminotadalafil (B). The bracketed numbers next to the *m*/*z* values indicate the charge state of the ions.

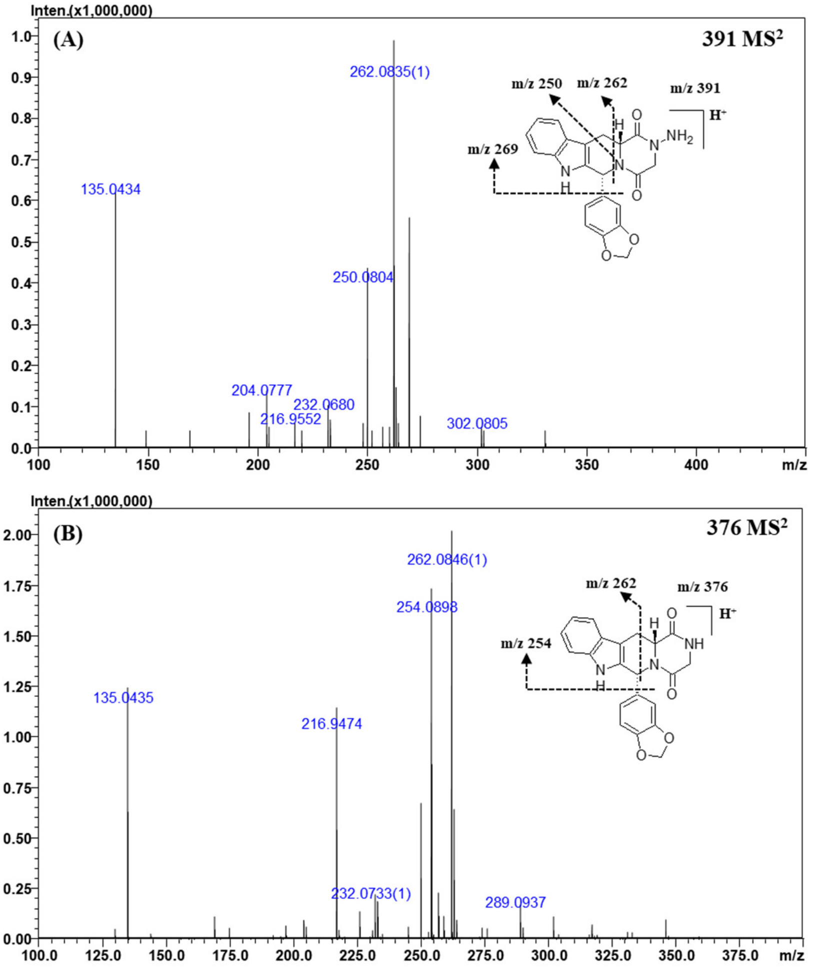


**Figure S14.** Representative MS[2] spectra and proposed fragmentation mechanisms of aminotadalafil (A) and demethyltadalafil (B). The bracketed numbers next to the *m*/*z* values indicate the charge state of the ions.

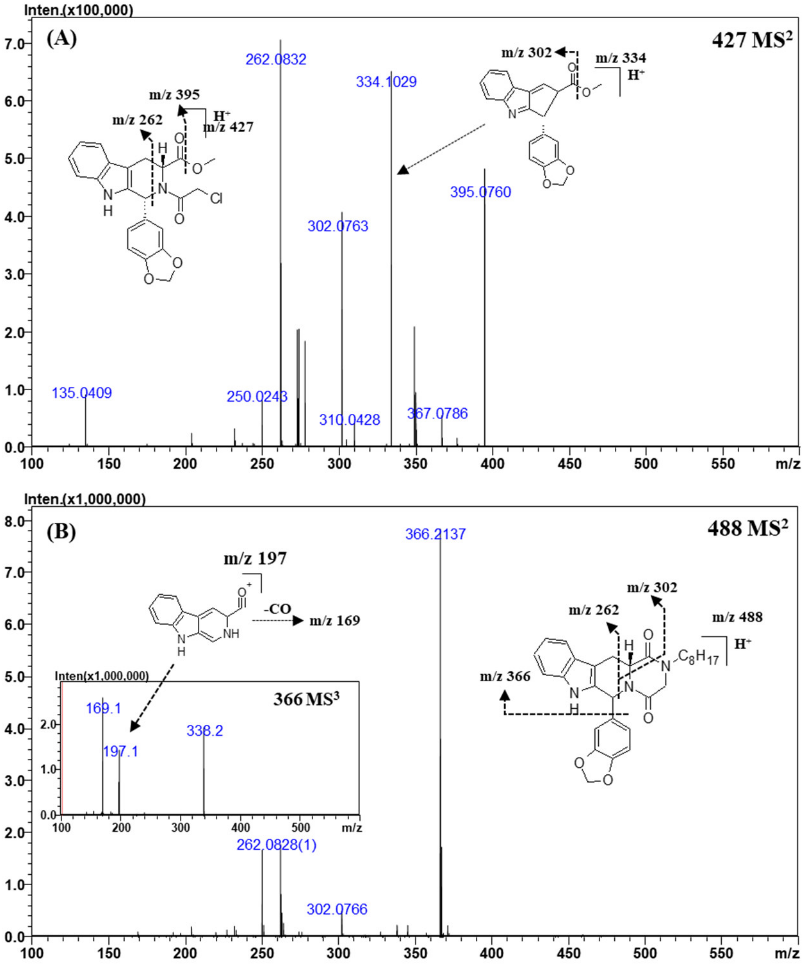


**Figure S15.** Representative MS$^n$ ($n$ = 2, 3) spectra and proposed fragmentation mechanisms of chloropretadalafil (A) and *N*-octyltadalafil (B). The bracketed numbers next to the *m*/*z* values indicate the charge state of the ions.

**Table S1.** Accurate masses and mass errors for fragment and precursor ions of PDE-5 inhibitors and their analogs.

| Compound | Elemental composition | Measured mass | Theoretical mass | $\Delta m$ (mDa) | Error (ppm) |
|---|---|---|---|---|---|
| Homosildenafil | $C_{23}H_{32}N_6O_4S$[a] | 245.1170[b] | 245.1176 | –0.6 | –2.45 |
| | $C_{17}H_{20}N_4O_2$ | 312.1576 | 312.1581 | –0.5 | –1.60 |
| | $C_{15}H_{16}N_4O_2$ | 284.1266 | 284.1268 | –0.2 | –0.70 |
| Dimethylsildenafil | $C_{23}H_{32}N_6O_4S$[a] | 245.1174[b] | 245.1176 | –0.2 | –0.82 |
| | $C_{17}H_{20}N_4O_2$ | 312.1558 | 312.1581 | –2.3 | –7.37 |
| | $C_{15}H_{16}N_4O_2$ | 284.1243 | 284.1268 | –2.5 | –8.80 |
| Thiohomosildenafil | $C_{23}H_{33}N_6O_3S_2$ | 253.1053[b] | 253.1061 | –0.8 | –3.32 |
| | $C_{17}H_{19}N_4OS$ | 328.1364 | 328.1352 | 1.2 | 3.66 |
| | $C_{15}H_{15}N_4OS$ | 300.1019 | 300.1039 | –2.0 | –6.66 |
| | $C_{14}H_{12}N_3O_3$ | 271.0920 | 271.0951 | –3.1 | –11.44 |
| Dimethylthiosildenafil | $C_{23}H_{33}N_6O_3S_2$[a] | 253.1060[b] | 253.1061 | –0.1 | –0.56 |
| | $C_{17}H_{19}N_4OS$ | 328.1336 | 328.1352 | –1.6 | –4.88 |
| | $C_{15}H_{15}N_4OS$ | 300.1046 | 300.1039 | 0.7 | 2.33 |
| | $C_{14}H_{12}N_3O_3$ | 271.0892 | 271.0951 | –5.9 | –21.76 |
| Benzylsildenafil | $C_{28}H_{34}N_6O_4S$[a] | 276.1239[b] | 276.1254 | –1.5 | –5.43 |
| | $C_{21}H_{28}N_6O_4S$ | 461.1970 | 461.1966 | 0.4 | 0.87 |
| | $C_{17}H_{20}N_4O_4S$ | 377.1272 | 377.1278 | –0.6 | –1.59 |
| | $C_{17}H_{18}N_4O_2$ | 311.1492 | 311.1503 | –1.1 | –3.54 |
| | $C_{15}H_{14}N_4O_2$ | 283.1191 | 283.1190 | 0.1 | 0.35 |
| | $C_{14}H_{14}N_4O$ | 255.1196 | 255.1240 | –4.4 | –17.25 |
| Carbodenafil | $C_{24}H_{32}N_6O_3$[a] | 227.1326[b] | 227.1341 | –1.5 | –6.60 |
| | $C_{16}H_{14}N_4O_3$ | 311.1119 | 311.1139 | –2.0 | –6.43 |
| | $C_{22}H_{28}N_6O_3$ | 213.1164[b] | 213.1184 | –2.0 | –9.38 |
| | $C_{20}H_{23}N_5O_3$ | 191.5971[b] | 191.5973 | –0.2 | –1.04 |
| | $C_{16}H_{14}N_4O_3$ | 156.0619[b] | 156.0606 | 1.3 | 8.33 |
| | $C_{18}H_{19}N_5O_3$ | 177.5830[b] | 177.5817 | 1.3 | 7.32 |
| Nor-neosildenafil | $C_{22}H_{29}N_5O_4S$[a] | 460.2011 | 460.2013 | –0.2 | –0.43 |
| | $C_{20}H_{25}N_5O_4S$ | 432.1686 | 432.1700 | –1.4 | –3.24 |
| | $C_{17}H_{20}N_4O_4S$ | 377.1267 | 377.1278 | –1.1 | –2.92 |
| | $C_{17}H_{18}N_4O_2$ | 311.1487 | 311.1503 | –1.6 | –5.14 |
| | $C_{15}H_{14}N_4O_2$ | 283.1180 | 283.1190 | –1.0 | –3.53 |
| | $C_{14}H_{14}N_4O$ | 255.1226 | 255.1240 | –1.4 | –5.49 |
| Cyclopentinafil | $C_{26}H_{36}N_6O_4S$[a] | 265.1322[b] | 265.1332 | –1.0 | –3.77 |
| | $C_{21}H_{28}N_6O_4S$ | 231.1009[b] | 231.1019 | –1.0 | –4.33 |
| | $C_{21}H_{28}N_6O_4S$ | 461.1951 | 461.1966 | –1.5 | –3.25 |
| | $C_{17}H_{20}N_4O_2$ | 312.1581 | 312.1581 | 0.0 | 0.00 |
| | $C_{19}H_{24}N_6O_4S$ | 217.0846[b] | 217.0863 | –1.7 | –7.83 |
| | $C_{15}H_{16}N_4O_2$ | 284.1225 | 284.1268 | –4.3 | –15.13 |
| Hydroxythiohomosildenafil | $C_{23}H_{32}N_6O_4S_2$[a] | 261.1011[b] | 261.1036 | –2.5 | –9.57 |
| | $C_{17}H_{20}N_4OS$ | 328.1340 | 328.1352 | –1.2 | –3.76 |
| | $C_{21}H_{25}N_6O_3S_2$ | 238.0813[b] | 238.0827 | –1.4 | –5.74 |
| | $C_{15}H_{16}N_4OS$ | 300.1028 | 300.1039 | –1.1 | –3.78 |
| Hydroxyhomosildenafil | $C_{23}H_{32}N_6O_5S$[a] | 253.1163[b] | 253.1150 | 1.3 | 5.14 |
| | $C_{17}H_{19}N_4O_2$ | 312.1576 | 312.1581 | –0.5 | –1.60 |
| | $C_{15}H_{15}N_4O_2$ | 284.1257 | 284.1268 | –1.1 | –3.87 |
| | $C_{21}H_{26}N_6O_4S$ | 230.0933[b] | 230.0941 | –0.8 | –3.48 |
| Chlorodenafil | $C_{19}H_{21}N_4O_3Cl$[a] | 389.1370 | 389.1375 | –0.5 | –1.28 |
| | $C_{17}H_{17}N_4O_3Cl$ | 361.1066 | 361.1062 | 0.4 | 1.11 |
| | $C_{15}H_{16}N_4O_2$ | 285.1339 | 285.1346 | –0.7 | –2.45 |
| Dichlorodenafil | $C_{19}H_{20}N_4O_2Cl_2$[a] | 407.1034 | 407.1036 | –0.2 | –0.49 |
| | $C_{17}H_{16}N_4O_2Cl_2$ | 379.0719 | 379.0723 | –0.4 | –1.08 |

| | | | | | |
|---|---|---|---|---|---|
| Hydroxychlorodenafil | $C_{19}H_{23}N_4O_3Cl$ [a] | 391.1538 | 391.1531 | 0.7 | 1.79 |
| | $C_{17}H_{19}N_4O_3Cl$ | 363.1217 | 363.1218 | –0.1 | –0.28 |
| | $C_{16}H_{16}N_4O_3$ | 313.1326 | 313.1295 | 3.1 | 9.90 |
| | $C_{15}H_{16}N_4O_2$ | 285.1346 | 285.1346 | 0.0 | 0.00 |
| Nitrodenafil | $C_{17}H_{19}N_5O_4$ [a] | 358.1506 | 358.1510 | –0.4 | –1.12 |
| | $C_{15}H_{15}N_5O_4$ | 330.1189 | 330.1197 | –0.8 | –2.42 |
| Udenafil | $C_{25}H_{36}N_6O_4S$ [a] | 259.1304 [b] | 259.1332 | –2.8 | –10.81 |
| | $C_{22}H_{30}N_6O_4S$ | 238.1087 [b] | 238.1097 | –1.0 | –4.20 |
| | $C_{15}H_{17}N_5O_4S$ | 364.1026 | 364.1074 | –4.8 | –13.18 |
| | $C_{15}H_{14}N_4O_3$ | 299.1133 | 299.1139 | –0.6 | –2.01 |
| | $C_{15}H_{14}N_4O_2$ | 283.1194 | 283.1190 | 0.4 | 1.41 |
| Hongdenafil | $C_{25}H_{34}N_6O_3$ [a] | 234.1398 [b] | 234.1419 | –2.1 | –8.97 |
| | $C_{23}H_{30}N_6O_3$ | 220.1223 [b] | 220.1262 | –3.9 | –17.72 |
| | $C_{21}H_{26}N_6O_3$ | 206.1049 [b] | 206.1106 | –5.7 | –27.66 |
| | $C_{17}H_{16}N_4O_3$ | 325.1335 | 325.1295 | 4.0 | 12.30 |
| | $C_{17}H_{18}N_4O_2$ | 311.1454 | 311.1503 | –4.9 | –15.75 |
| | $C_{16}H_{16}N_4O_2$ | 297.1330 | 297.1346 | –1.6 | –5.38 |
| Piperidinohongdenafil | $C_{24}H_{31}N_5O_3$ [a] | 438.2501 | 438.2500 | 0.1 | 0.23 |
| | $C_{19}H_{20}N_4O_3$ | 353.1601 | 353.1608 | –0.7 | –1.98 |
| | $C_{18}H_{20}N_4O_3$ | 341.1592 | 341.1608 | –1.6 | –4.69 |
| | $C_{17}H_{16}N_4O_3$ | 325.1293 | 325.1295 | –0.2 | –0.62 |
| Demethylhongdenafil | $C_{24}H_{32}N_6O_3$ [a] | 227.1331 [b] | 227.1341 | –1.0 | –4.40 |
| | $C_{22}H_{28}N_6O_3$ | 213.1165 [b] | 213.1184 | –1.9 | –8.92 |
| | $C_{17}H_{18}N_4O_2$ | 311.1455 | 311.1503 | –4.8 | –15.43 |
| | $C_{16}H_{16}N_4O_2$ | 297.1330 | 297.1346 | –1.6 | –5.38 |
| | $C_{22}H_{28}N_6O_3$ | 213.1165 [b] | 213.1184 | –1.9 | –8.92 |
| | $C_{19}H_{21}N_5O_3$ | 184.5896 | 184.5895 | 0.1 | 0.54 |
| Hydroxyhongdenafil | $C_{25}H_{34}N_6O_4$ [a] | 242.1372 [b] | 242.1394 | –2.2 | –9.09 |
| | $C_{23}H_{30}N_6O_4$ | 228.1228 [b] | 228.1237 | –0.9 | –3.95 |
| | $C_{23}H_{28}N_6O_3$ | 219.1171 [b] | 219.1184 | –1.3 | –5.93 |
| | $C_{17}H_{18}N_4O_3$ | 327.1440 | 327.1452 | –1.2 | –3.67 |
| | $C_{17}H_{20}N_4O_2$ | 312.1558 | 312.1581 | –2.3 | –7.37 |
| | $C_{15}H_{16}N_4O_2$ | 284.1243 | 284.1268 | –2.5 | –8.80 |
| Oxohongdenafil | $C_{25}H_{32}N_6O_4$ [a] | 241.1306 [b] | 241.1315 | –0.9 | –3.73 |
| | $C_{22}H_{27}N_5O_3$ | 205.6100 [b] | 205.6130 | –3.0 | –14.59 |
| | $C_{21}H_{25}N_5O_3$ | 198.6032 [b] | 198.6051 | –1.9 | –9.57 |
| | $C_{20}H_{23}N_5O_3$ | 191.5963 [b] | 191.5973 | –1.0 | –5.22 |
| | $C_{19}H_{21}N_5O_3$ | 184.5892 [b] | 184.5895 | –0.3 | –1.63 |
| Desulfovardenafil | $C_{17}H_{20}N_4O_2$ [a] | 313.1648 | 313.1659 | –1.1 | –3.51 |
| | $C_{15}H_{16}N_4O_2$ | 285.1348 | 285.1346 | 0.2 | 0.70 |
| | $C_8H_{10}N_2O$ | 151.0876 | 151.0866 | 1.0 | 6.62 |
| Nor-neovardenafil | $C_{18}H_{20}N_4O_4$ [a] | 357.1546 | 357.1557 | –1.1 | –3.08 |
| | $C_{16}H_{16}N_4O_4$ | 329.1237 | 329.1244 | –0.7 | –2.13 |
| | $C_8H_{10}N_2O$ | 151.0877 | 151.0866 | 1.1 | 7.28 |
| | $C_{15}H_{16}N_4O_2$ | 285.1339 | 285.1346 | –0.7 | –2.45 |
| Pseudovardenafil | $C_{22}H_{29}N_5O_4S$ [a] | 460.2016 | 460.2013 | 0.3 | 0.65 |
| | $C_{20}H_{25}N_5O_4S$ | 432.1722 | 432.1700 | 2.2 | 5.09 |
| | $C_{17}H_{20}N_4O_4S$ | 377.1273 | 377.1278 | –0.5 | –1.33 |
| | $C_{17}H_{20}N_4O_2$ | 312.1564 | 312.1581 | –1.7 | –5.45 |
| | $C_{15}H_{16}N_4O_2$ | 284.1255 | 284.1268 | –1.3 | –4.58 |
| | $C_8H_{10}N_2O$ | 151.0870 | 151.0866 | 0.4 | 2.65 |
| Acetylvardenafil | $C_{25}H_{34}N_6O_3$ [a] | 234.1407 [b] | 234.1419 | –1.2 | –5.13 |
| | $C_{23}H_{30}N_6O_3$ | 220.1226 [b] | 220.1262 | –3.6 | –16.35 |
| | $C_{21}H_{26}N_6O_3$ | 206.1135 [b] | 206.1106 | 2.9 | 14.07 |
| | $C_{19}H_{21}N_5O_3$ | 184.5904 [b] | 184.5895 | 0.9 | 4.88 |

| | | | | | |
|---|---|---|---|---|---|
| | $C_{15}H_{16}N_4O_2$ | 284.1256 | 284.1268 | –1.2 | –4.14 |
| | $C_{17}H_{16}N_4O_3$ | 325.1332 | 325.1295 | 3.7 | 11.38 |
| | $C_{16}H_{16}N_4O_2$ | 297.1333 | 297.1346 | –1.3 | –4.38 |
| | $C_8H_{10}N_2O$ | 151.0876 | 151.0866 | 1.0 | 6.62 |
| Hydroxyvardenafil | $C_{23}H_{32}N_6O_5S$ [a] | 253.1159 [b] | 253.1150 | 0.9 | 3.56 |
| | $C_{17}H_{19}N_4O_2$ | 312.1575 | 312.1581 | –0.6 | –1.92 |
| | $C_{15}H_{15}N_4O_2$ | 284.1265 | 284.1268 | –0.3 | –1.06 |
| | $C_{21}H_{26}N_6O_4S$ | 230.0938 [b] | 230.0941 | –0.3 | –1.30 |
| | $C_8H_{10}N_2O$ | 151.0866 | 151.0866 | 0.0 | 0.00 |
| Acetaminotadalafil | $C_{23}H_{20}N_4O_5$ [a] | 433.1496 | 433.1506 | –1.0 | –2.31 |
| | $C_{21}H_{18}N_4O_4$ | 391.1433 | 391.1401 | 3.2 | 8.18 |
| | $C_{16}H_{14}N_4O_3$ | 311.1121 | 311.1139 | –1.8 | –5.79 |
| | $C_{14}H_{12}N_4O_2$ | 269.1029 | 269.1033 | –0.4 | –1.49 |
| | $C_{17}H_{11}NO_2$ | 262.0864 | 262.0863 | 0.1 | 0.38 |
| | $C_{13}H_9N_3O$ | 224.0781 | 224.0818 | –3.7 | –16.51 |
| | $C_{11}H_8N_2$ | 169.0780 | 169.0760 | 2.0 | 11.83 |
| Aminotadalafil | $C_{21}H_{18}N_4O_4$ [a] | 391.1399 | 391.1401 | –0.2 | –0.51 |
| | $C_{14}H_{12}N_4O_2$ | 269.1017 | 269.1033 | –1.6 | –5.95 |
| | $C_{17}H_{11}NO_2$ | 262.0859 | 262.0863 | –0.4 | –1.53 |
| | $C_{16}H_{11}NO_2$ | 250.0849 | 250.0863 | –1.4 | –5.60 |
| | $C_{11}H_8N_2$ | 169.0771 | 169.0760 | 1.1 | 6.51 |
| | $C_8H_6O_2$ | 135.0451 | 135.0441 | 1.0 | 7.40 |
| Demethyltadalafil | $C_{21}H_{17}N_3O_4$ [a] | 376.1287 | 376.1292 | –0.5 | –1.33 |
| | $C_{17}H_{11}NO_2$ | 262.0856 | 262.0863 | –0.7 | –2.67 |
| | $C_{14}H_{11}N3O_2$ | 254.0912 | 254.0924 | –1.2 | –4.72 |
| | $C_{16}H_9NO$ | 232.0744 | 232.0757 | –1.3 | –5.60 |
| | $C_{15}H_9N$ | 204.0797 | 204.0808 | –1.1 | –5.39 |
| | $C_{11}H_8N_2$ | 169.0751 | 169.0760 | –0.9 | –5.32 |
| | $C_8H_6O_2$ | 135.0449 | 135.0441 | 0.8 | 5.92 |
| Chloropretadalafil | $C_{22}H_{19}N_2O_5Cl$ [a] | 427.1039 | 427.1055 | –1.6 | –3.75 |
| | $C_{21}H_{15}N_2O_4Cl$ | 395.0788 | 395.0793 | –0.5 | –1.27 |
| | $C_{20}H_{16}N_2O_4$ | 349.1168 | 349.1183 | –1.5 | –4.30 |
| | $C_{20}H_{15}NO_4$ | 334.1063 | 334.1074 | –1.1 | –3.29 |
| | $C_{19}H_{11}NO_3$ | 302.0812 | 302.0812 | 0.0 | 0.00 |
| | $C_{17}H_{11}NO_2$ | 262.0849 | 262.0863 | –1.4 | –5.34 |
| *N*-Octyltadalafil | $C_{29}H_{33}N_3O_4$ [a] | 488.2540 | 488.2544 | –0.4 | –0.82 |
| | $C_{22}H_{27}N_3O_2$ | 366.2170 | 366.2176 | –0.6 | –1.64 |
| | $C_{19}H_{11}NO_3$ | 302.0778 | 302.0812 | –3.4 | –11.26 |
| | $C_{17}H_{11}NO_2$ | 262.0878 | 262.0863 | 1.5 | 5.72 |
| | $C_{21}H_{27}N_3O$ | 338.2207 | 338.2227 | –2.0 | –5.91 |
| | $C_{12}H_8N_2O$ | 197.0710 | 197.0709 | 0.1 | 0.51 |
| | $C_{11}H_8N_2$ | 169.0770 | 169.0760 | 1.0 | 5.91 |

[a] Precursor ion; [b] Multiple charged protonated ion $[M+2H]^{2+}$.

**Table S2.** The structures of PDE-5 inhibitors and their analogues.

| Backbone of the structure | Name of compound | R1 | R2 |
|---|---|---|---|
| 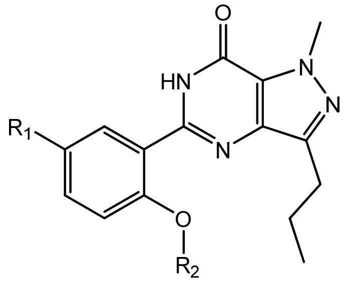 | Sildenafil | 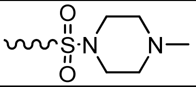 | $C_2H_5$ |
| | Homosildenafil | 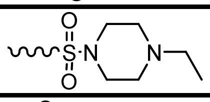 | |
| | Hydroxyhomosildenafil | 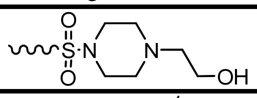 | |
| | Dimethylsildenafil | 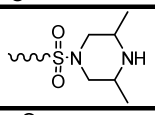 | |
| | Cyclopentinafil | 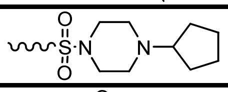 | |
| | Norneosildenafil | 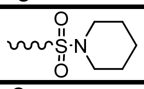 | |
| | Benzylsildenafil | 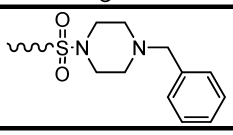 | |
| | Carbodenafil | 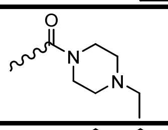 | |
| | Hondenafil | 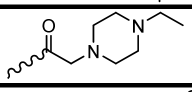 | |
| | Hydroxyhongdenafil | 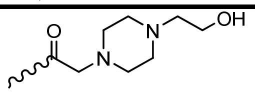 | |
| | Demethylhongdenafil | 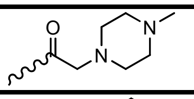 | |
| | Piperidinohongdenafil | 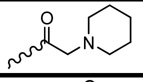 | |
| | Oxohongdenafil | 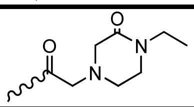 | |
| | Chlorodenafil | 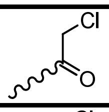 | |
| | Hydroxychlorodenafil | 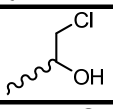 | |
| | Nitrodenafil | 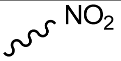 | |
| | Dichlorodenafil | 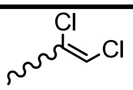 | |
| | Udenafil | 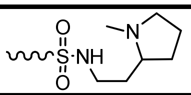 | $C_3H_7$ |
| | Thiosildenafil | 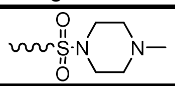 | $C_2H_5$ |

| | | | |
|---|---|---|---|
| 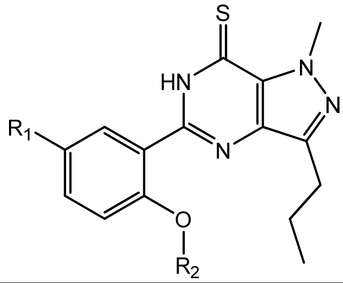 | Thiohomosildenafil | 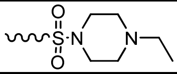 | |
| | Hydroxythiohomosildenafil | 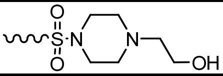 | |
| | Dimethylthiosildenafil | 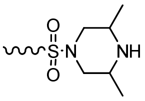 | |
| 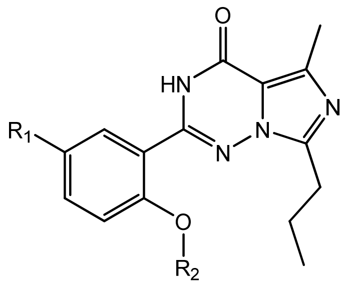 | Vardenafil | 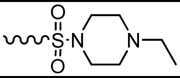 | |
| | Acetylvardenafil | 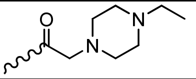 | |
| | Hydroxyvardenafil | 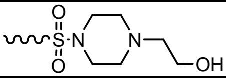 | $C_2H_5$ |
| | norneovardenafil | 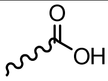 | |
| | Pseudovardenafil | 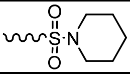 | |
| | Desulfovardenafil | H | |
| 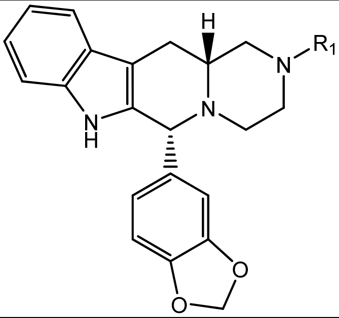 | Tadalafil | $CH_3$ | |
| | Aminotaldalfil | $NH_2$ | |
| | Acetaminotadalafil | 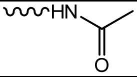 | |
| | Demethyltadalafil | H | |
| | *N*-octyltadalafil | $C_8H_{17}$ | - |
| 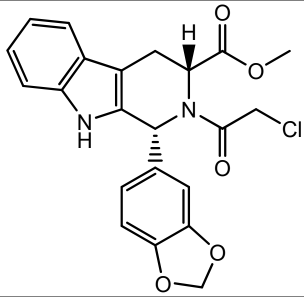 | Chloropretadalafil | - | |
| 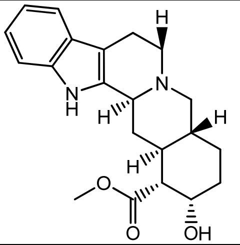 | Yohimbine | - | - |
| 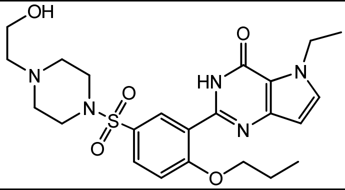 | Mirodenafil | - | - |

| | | | |
|---|---|---|---|
| 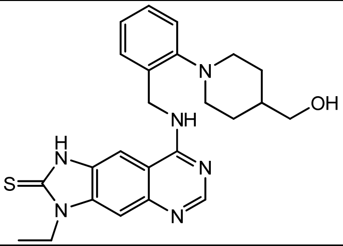 | Thioquinapiperifil | - | - |
| 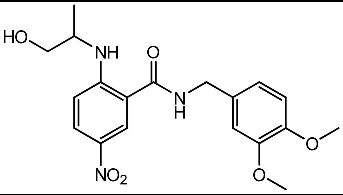 | Xanthoanthrafil | | |
| 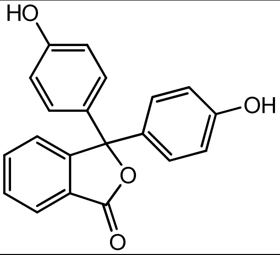 | Phenolphtalein (IS) | - | - |

*All analytes contain guanosine like structure as a pharmacophore.
